# Pathogen Propagation Model with Superinfection in Vegetatively Propagated Plants on Lattice Space

**DOI:** 10.1371/journal.pone.0154883

**Published:** 2016-05-05

**Authors:** Yuma Sakai, Takenori Takada

**Affiliations:** 1 Graduate School of Environmental Science, Hokkaido University, Sapporo, Hokkaido, Japan; 2 Graduate School of Environmental Earth Science, Hokkaido University, Sapporo, Hokkaido, Japan; National University of Ireland - Galway, IRELAND

## Abstract

Many clonal plants have two reproductive patterns, seed propagation and vegetative propagation. By vegetative propagation, plants reproduce the genetically identical offspring with a low mortality, because resources are supplied from the other individuals through interconnected ramets at vegetative-propagated offspring. However, the ramets transport not only resources but also systemic pathogen. Pathogens evolve to establish and spread widely within the plant population. The superinfection, which is defined as the ability that an established pathogen spreads widely by infecting to already-infected individuals with other strains of a pathogen, is important to the evolution of pathogens. We examine the dynamics of plant reproduction and pathogen propagation considering spatial structure and the effect of superinfection on genetic diversity of pathogen by analysis of several models, 1-strain and multiple-strain models, on two-dimensional square lattice. In the analysis of 1-strain model, we derive equilibrium value by mean-field approximation and pair approximation, and its local stability by Routh-Hurwitz stability criterion. In the multiple-strain models, we analyze the dynamics by numerical simulation of mean-field approximation, pair approximation and Monte Carlo simulation. Through the analyses, we show the effect of parameter values to dynamics of models, such as transition of dominant strain of pathogen, competition between plants and pathogens and density of individuals. As a result, (i) The strain with intermediate cost becomes dominant when both superinfection rate and growth rate are low. (ii) The competition between plants and pathogens occurs in the phase of coexistence of various strains by pair approximation and Monte Carlo simulation. (iii) Too high growth rate leads to the decrease of plant population in all models. (iv) Pathogens are easy to maintain their genetic diversity with low superinfection rate. However, if they do not superinfect, the maintenance becomes difficult. (v) When growth rate of plant is low, individuals are very influenced by distant individuals.

## Introduction

Many clonal plants have two reproductive patterns, seed propagation and vegetative propagation (i.e. clonal growth). By seed propagation, plants reproduce genetically different offspring with a high mortality, because seed-propagated offspring are not supported by their parents (no physical connection). By contrast, in vegetative propagation, plants reproduce genetically identical offspring with a lower mortality, because resources are supplied from the other individuals through interconnected ramets at vegetative-propagated offspring [1, 2]. However, if some of systemic pathogens invade the population, the pathogens spread rapidly within the population, because ramets transport not only resources, but also pathogens [3].

According to Stuefer et al. (2004), pathogens have diverse negative effects on plants, such as lethality or severe damage. For example, they lead to deformation of leaves [4], reduction the growth rate [5–7], change of the growth form of plants [8, 9] and effectively block plant reproduction [10–12]. Thus, the possibility that the plant population suffers serious damage through effect of a pathogen is high, and plants have evolved to prevent the spread of infection, such as escaping by increasing their growth rate [8].

Contrastingly, pathogens have evolved to establish and spread widely within the plant population. A diversity of infections, e.g. superinfection, plays an important role in the evolution of pathogens [13–15]. Superinfection is defined as the ability of an established pathogen to spread widely by infecting already-infected individuals with other strains of a pathogen (secondary infection) [14]. Thus, competition among different strains of a pathogen occurs and leads to increased fitness of the pathogens relative to single infection [16].

There are several theoretical studies pertinent to evolution of pathogen virulence with superinfection [17–23]. They defined that the already-infected individual is supreinfected and taken over by other strain of pathogen with higher virulence, thus strains do not share in a host. Additionally, most of them assumed that there is trade-off between the infection rate and the virulence of the pathogen, and considered the evolution of virulence in a host population by the host-parasite model. If hosts are long-lived, pathogens should get a long-term benefit from the plants by a low infection rate. However, if hosts are short-lived, the infection rate is supposed to evolve to a higher value, because pathogens should propagate quickly in a new host before the death of the hosts from the high infection rate. In addition, previous studies analyzed the evolution of virulence in the two cases where a host is either infected by only one strain of a pathogen (single infection) or by several strains (superinfection) of a pathogen. As a result, in the model of single infection, the virulence evolves towards an intermediate value. In the other case, it evolves towards higher virulence compared with single infection. However, they did not consider the spatial structures, such as the configuration of ramets in plant population.

Thus, we focus on the spatial structures, which play an important role in evolution of both plants and pathogens. The interaction between a plant and a pathogen depends on the spatiotemporal dynamics of pathogen dispersal, the genetic diversity of the host plant population and the spatial positioning of ramets [24, 25]. According to Koubek and Herben (2008), there are several defense reactions against systemic pathogens in clonal plant, such as (i) increasing the growth speed of the plant [8, 9, 26–28], (ii) dispersing the risk of infection and spread of pathogen by splitting the physical connection of ramets [7, 29], and (iii) detaching the infected ramets or tissues deliberately [30]. Additionally it is expected that features of the host would assist local pathogen transmission and evolution of the pathogen to lower levels of virulence [21], because clonal growth increases the probability of finding susceptible hosts in the vicinity of the initially infected host. However, they did not consider the superinfection event.

To explain these dynamics, we constructed a model based on the contact process (CP) [31], especially the two stage contact process (TCP) [32, 33], in mathematical models. These are simple models that express the dynamics of the contact infection process. The CP assumes only two states, healthy and infected, and healthy individuals are infected by only neighboring infected individuals. By contrast, TCP assumes three states, such as empty, healthy and infected, and healthy individuals reproduce new offspring and infected individuals increase only by transmission to healthy ones. Therefore, we adopted TCP to express not only the infection process, but also the plant growth process. In addition, we analyzed this model on the lattice space for simplicity. However, an explicit solution was not obtained in TCP, because it is too difficult to solve analytically. Thus, we performed an approximation by applying the mean-field approximation (MA) and the pair approximation (PA) to discuss the behavior of the system analytically. MA is the simplest approximation method, which takes no account of the effect of other sites. PA assumes that the effects of distant sites will be less important than those of the nearest neighbor sites. Therefore, this approximation is very useful to analyze the effect of local connections. Several studies have applied PA to TCP [34–36]. Satō et al. (1994) analyzed TCP in detail using PA; however, they could not determine the stability of the epidemic equilibrium (coexistence of healthy and infected individuals) analytically. Satulovsky and Tomé (1994) studied a predator-prey system using an improved TCP that had similar transition rules to our model, and obtained the analytical result with respect to the coexistence equilibrium. However, we did not use their model to express the superinfection process directly because they assumed only one predator species (not many). Haraguchi and Sasaki (2000) considered mutants of pathogens, which have additional mortality, based on TCP and analyzed the ESS (Evolutionarily Stable Strategy) of the mortality and transmission rate by computer simulation. However they did not include the superinfection event in propagation process of pathogens. Thus, further modification of the models is needed to express the pathogen spread process, including superinfection.

In this paper, we explain the plant reproduction and pathogen propagation dynamics considering special structures (using lattice structured space) and the effect of superinfection events on genetic diversity of pathogens using several models, the 1-strain and multiple-strain models. In the analysis of the 1-strain model, we derived an equilibrium value by MA and PA, and its local stability by the Routh—Hurwitz stability criterion. In the multiple-strain models, we analyzed the dynamics by numerical simulation using MA, PA and Monte Carlo simulation (MCS). Through these analyses, we showed the effect of parameter values on the dynamics of the models, such as density of individuals, transition of a dominant strain of a pathogen, and competition between plants and pathogens. As a result, the superinfection event leads to the polymorphism of pathogen strains in the host plant population, the dominant strain changes depending on plant and pathogen ability; and the competition is observed in particular parameter range. Thus, the plant and pathogen affect in the course of evolution mutually. In addition, when the growth rate of a plant is low, our present model is largely affected by the spatial structure.

## Analysis

We constructed a model of plant growth and pathogen propagation processes, including superinfection. In the model, we assume that there is a single species of plant and multiple strains of a pathogen, and that a healthy plant individual is infected by a strain of the pathogen and an already-infected plant is superinfected by other strains of the pathogen. The dynamics of our model is a continuous Markov process on a lattice space, and the states of each site, the transition rate and mortality of each state are represented by a vector ***Ω*** = (*σ*_0_, *σ*_1_, *σ*_2_,…,*σ*_*n*_), ***B*** = (*β*_*σ*_0__, *β*_*σ*_1__, *β*_*σ*_2__,…,*β*_*σ*_*n*__) and ***D*** = (*d*_*σ*_0__, *d*_*σ*_1__, *d*_*σ*_2__,…,*d*_*σ*_*n*__), respectively, where the total number of states is *n*+1. Let *ρ*_*σ*_*i*__(*t*) be the probability that a randomly chosen site has state *σ*_*i*_ at time *t*, in other words, *ρ*_*σ*_*i*__(*t*) means the global density of the site with state *σ*_0_ = “0”, *σ*_1_ = “S”, and *σ*_*i*+1_ = “I_*i*_”(*i* = 1, 2,…,*n* − 1), which mean the empty, susceptible (healthy) individual, and infected individual by a pathogen with *i*-strains, respectively. In addition, we assumed that the already-infected individuals with *i*-strain (“I_*i*_“) are superinfected (and taken over) by the more virulent *j*-strain than *i*-strain, because the strain with higher virulence often wins within-host competition [21, 37]. From the definition of *d*_0_ and *β*_0_, *d*_0_ = 0 and $\beta_{0}=\frac{1}{n}\sum_{i=1}^{n}d_{\sigma_{i}}$ because the empty site does not die and transition to “0” means death of healthy and infected individuals.

We configured four demographic processes: (i) the plant growth process, (ii) the first infection process, (iii) the superinfection process and (iv) the death process. The growth process is represented by transition from state “0” to “S”, which means that plants grow their ramets into an open area (i.e. an empty site is occupied by a healthy individual). The first infection process is represented by transition from state “S” to “I_*i*_”, which means healthy individuals are infected by pathogens of the *i*-strain. The superinfection process is represented by transition from state “I_*i*_” to “I_*j*_” (*i* > *j*). The death process is represented by transition from “S” or “I_*i*_” to “0” which represent the death of healthy and infected individuals from natural causes and virulence of the pathogen, respectively. In addition, infected individuals are not able to recover to a healthy one. Thus, we describe these processes by the following notation, which is often used to explain the TCP.

$$\begin{array}{c} \begin{array}{cccccc} \text{(i)} & 0 & \to & S & \text{at}\;\text{rate} & \frac{\beta_{S}n\left(S\right)}{z}\\ \text{(ii)} & S & \to & I_{i} & \text{at}\;\text{rate} & \frac{\beta_{I_{i}}n\left(I_{i}\right)}{z}\\ \text{(iii)} & I_{i} & \to & I_{j} & \text{at}\;\text{rate} & s\frac{\beta_{I_{j}}n\left(I_{j}\right)}{z}\\ \text{(iv)} & S,\;I_{i} & \to & 0 & \text{at}\;\text{rate} & d_{S},\;d_{I_{i}} \end{array}\;\left(\right) \end{array}$$ (TP)

Parameter *n*(*σ*_*i*_) is the number of *σ*_*i*_-sites in the nearest neighbor of focal sites, *z* is the number of nearest-neighbor sites (e.g. *z* = 4 for von Neumann neighborhood on the two-dimensional square lattice), and *s* is the superinfection rate. Thus $s\beta_{I_{j}}$ describes the proportion of superinfection to first infection [18]. The growth or infection event occurs at a rate proportional to the number of the healthy or infected state in nearest-neighbor sites, respectively.

Here, let *q*_*σ*_*j*_/*σ*_*i*__(*t*) be the conditional probability that a randomly chosen nearest neighbor of a *σ*_*i*_-site is a *σ*_*j*_-site. In particular, *q*_*σ*_*i*_/*σ*_*i*__ means the local density of *σ*_*i*_-site. *P*_*σ*_*i*_*σ*_*j*__(*t*) is the probability that a randomly chosen site has state *σ*_*i*_ and a randomly chosen nearest-neighbor site has state *σ*_*j*_ at time *t*. These variables have the following relationship [34, 38].

$$\begin{array}{c} P_{\sigma_{i}\sigma_{j}}=\rho_{\sigma_{i}}q_{\sigma_{j}/\sigma i}. \end{array}$$ (1)

Thus, we can describe the following set of master equations, which is referred to as the general model (GM) from the above dynamics.

$$\begin{array}{c} \begin{array}{cc} {\dot{P}}_{00}= & 2\left(\sum_{i=1}^{n-1}d_{I_{i}}P_{I_{k}0}+d_{S}P_{S0}-\frac{\beta_{S}\left(z-1\right)q_{S/00}}{z}P_{00}\right),\\ {\dot{P}}_{\text{S}0}= & \frac{\beta_{S}\left(z-1\right)q_{S/00}}{z}P_{00}+d_{S}P_{SS}-\frac{\beta_{S}}{z}P_{S0}-\frac{\beta_{S}\left(z-1\right)q_{S/0S}}{z}P_{S0}\\ & -d_{S}P_{S0}+\sum_{i=1}^{n-1}\left[d_{I_{i}}P_{SI_{i}}-\frac{\beta_{I_{i}}\left(z-1\right)q_{I_{i}/S0}}{z}P_{S0}\right],\\ {\dot{P}}_{\text{SS}}= & 2\left(\frac{\beta_{S}}{z}P_{S0}+\frac{\beta_{S}\left(z-1\right)q_{S/0S}}{z}P_{S0}-\sum_{i=1}^{n-1}\frac{\beta_{I_{i}}\left(z-1\right)q_{I_{i}/SS}}{z}P_{SS}-d_{S}P_{SS}\right),\\ {\dot{P}}_{{\text{I}}_{i}0}= & \frac{\beta_{I_{i}}\left(z-1\right)q_{I_{i}/S0}}{z}P_{S0}+d_{S}P_{SI_{i}}-\frac{\beta_{S}\left(z-1\right)q_{S/0I_{i}}}{z}P_{I_{i}0}+\sum_{j=1}^{n-1}d_{I_{j}}P_{I_{i}I_{j}}-d_{I_{i}}P_{I_{i}0}\\ & +s\left(\sum_{j=i+1}^{n-1}\frac{\beta_{I_{i}}\left(z-1\right)q_{I_{i}/I_{j}0}}{z}P_{I_{j}0}-\sum_{j=1}^{i-1}\frac{\beta_{I_{j}}\left(z-1\right)q_{I_{j}/I_{i}0}}{z}P_{I_{i}0}\right),\\ {\dot{P}}_{\text{S}{\text{I}}_{i}}= & \frac{\beta_{S}\left(z-1\right)q_{S/0I_{i}}}{z}P_{0I_{i}}+\frac{\beta_{I_{i}}\left(z-1\right)q_{I_{i}/SS}}{z}P_{SS}-\frac{\beta_{I_{i}}}{z}P_{SI_{i}}-\sum_{j=1}^{n-1}\frac{\beta_{I_{j}}\left(z-1\right)q_{I_{j}/SI_{i}}}{z}P_{SI_{i}}\\ & -\left(d_{S}+d_{I_{i}}\right)P_{SI_{i}}+s\left(\sum_{j=i+1}^{n-1}\frac{\beta_{I_{i}}\left(z-1\right)q_{I_{i}/I_{j}S}}{z}P_{SI_{j}}-\sum_{j=1}^{i-1}\frac{\beta_{I_{j}}\left(z-1\right)q_{I_{j}/I_{i}S}}{z}P_{SI_{i}}\right),\\ {\dot{P}}_{{\text{I}}_{i}{\text{I}}_{j}}= & \frac{\beta_{I_{i}}\left(z-1\right)q_{I_{i}/SI_{j}}}{z}P_{SI_{j}}+\frac{\beta_{I_{j}}\left(z-1\right)q_{I_{j}/SI_{i}}}{z}P_{I_{i}S}-\left(d_{I_{i}}+d_{I_{j}}\right)P_{I_{i}I_{j}}\\ & +s\left(\sum_{k=i+1}^{n-1}\frac{\beta_{I_{i}}\left(z-1\right)q_{I_{i}/I_{k}I_{j}}}{z}P_{I_{k}I_{j}}+\sum_{k=j+1}^{n}\frac{\beta_{I_{j}}\left(z-1\right)q_{I_{j}/I_{k}I_{i}}}{z}P_{I_{i}I_{k}}\right.\\ & \left.-\sum_{k=1}^{i-1}\frac{\beta_{I_{k}}\left(z-1\right)q_{I_{k}/I_{i}I_{j}}}{z}P_{I_{i}I_{j}}-\sum_{k=1}^{j-1}\frac{\beta_{I_{k}}\left(z-1\right)q_{I_{k}/I_{j}I_{i}}}{z}P_{I_{i}I_{j}}-\frac{\beta_{I_{j}}}{z}P_{I_{i}I_{j}}\right)\;\left(i>j\right),\\ {\dot{P}}_{{\text{I}}_{i}{\text{I}}_{i}}= & 2\left[\frac{\beta_{I_{i}}}{z}P_{SI_{i}}+\frac{\beta_{I_{i}}\left(z-1\right)q_{I_{i}/SI_{i}}}{z}P_{SI_{i}}-d_{I_{i}}P_{I_{i}I_{i}}\right.\\ & \left.+s\left(\sum_{j=i+1}^{n-1}\left(\frac{\beta_{i}}{z}P_{I_{i}I_{j}}+\frac{\beta_{I_{i}}\left(z-1\right)q_{I_{i}/I_{j}I_{i}}}{z}P_{I_{j}I_{i}}\right)-\sum_{j=1}^{i-1}\frac{\beta_{I_{j}}\left(z-1\right)q_{I_{j}/I_{i}I_{i}}}{z}P_{I_{i}I_{i}}\right)\right]. \end{array} \end{array}$$ (2)

Here, for example, in the right side of the fifth equation in the set (i.e. differential equation of $P_{SI_{i}}$), the first term describes the transition from $P_{0I_{i}}$ to $P_{SI_{i}}$, which means healthy individuals reproduce their offspring to empty sites (state 0 → S). In this term, the transition rate is determined by TP(i), *n*(S) is $(z-1)q_{S/0I_{i}}$. Transition begins from $P_{0I_{i}}$; therefore, one of the nearest-neighbor sites of the 0-state site is state I_*i*_, and at least one of the other nearest-neighbor sites of the 0-state site (in (*z*−1) sites) should be S to transition from 0 to S. Thus, the probability is $q_{S/0I_{i}}$, and the expectation of *n*(S) is equal to $(z-1)q_{S/0I_{i}}$. In subsequent terms, the *n*(*σ*) (*σ* ∈ *Ω*) is obtained in a similar process, except the third term.

The second and third terms mean that healthy individuals are infected by pathogens with *i*-strain (state S → I_*i*_). These terms describe the transition from *P*_*SS*_ to $P_{SI_{i}}$ and from $P_{SI_{i}}$ to $P_{I_{i}I_{i}}$, respectively, and the transition rate of these terms is determined by TP(ii). In particular, the value of *n*(I_*i*_) in the third term is equal to 1. The transition begins from $P_{SI_{i}}$; therefore, there is already a I_*i*_-state site in nearest-neighbor sites of S-state site. Here, the case that the state of other sites in the nearest-neighbor sites is also I_*i*_ is included in the fourth term.

The fourth term means that healthy individuals are infected by pathogens with *j*-strain (state S → I_*j*_). This term describes the transition from $P_{SI_{i}}$ to $P_{I_{j}I_{i}}$ (*i* ∈ *j*), and sums the transition rates in respect to all strains (from I_1_ to I_*n*−1_).

The fifth term means the death of healthy or infected individuals (state S, I_*i*_ → 0). The term describes the transition from $P_{SI_{i}}$ to $P_{0I_{i}}$ or *P*_S0_ and the transition rate of each process is determined by TP(iv).

The last term means that the already-infected individuals are superinfected by other strains of the pathogen (e.g. state I_2_ → I_1_). The first and second terms in parentheses describe the transition from $P_{SI_{j}}$ to $P_{SI_{i}}$ and from $P_{SI_{i}}$ to $P_{SI_{j}}$ (*j* ≠ *i*), respectively, and the transition rate of these term is determined by TP(iii). The first term means that the infecting pathogens superinfect already-infected individuals with another strain, and the second term means that an already-infected individual is superinfeced by another strain of the pathogen. Thus, the range of summation in the first term is from *i*+1 to *n*−1 and in the second term is from 1 to *i*−1 from our assumption.

## Results

In the subsequent sections, we introduce a new parameter *m*_*i*_ (we refer to the parameter as mortality cost in this paper) defined as $\beta_{I_{i}}/d_{I_{i}}$ for *n* − 1 strains ($m_{i}:=\beta_{I_{i}}/d_{I_{i}}$, *m*_1_ < *m*_2_ < ⋯ < *m*_*n*−1_), and set $d_{I_{i}}=1$ (∀I_i_ ∈ Ω) to standardize the parameter, for ease of analysis. The mortality cost means the expectation of the number of new infected-individual produced during a lifetime of an infected-individual, thus, higher virulent strain has lower mortality cost. Consequently, already-infected individuals are superinfected by the strains with lower mortality costs. In addition, we set *d*_S_ ≈ 0, because the plant mortality is generally smaller than plant growth rate in clonal plant (i.e. the plants are long lived).

### 1-strain model

Initially, we analyzed the simplest case (*n* = 2) in GM by MA and PA. The state of each site was denoted by *σ*_*i*_ ∈ ***S*** ≡ {0, S, I}(I: = I_1_) from the assumption of only one strain of pathogen. Therefore, we obtained the following set of master equations to rewrite Eq (2).

$$\begin{array}{c} \begin{array}{cc} {\dot{P}}_{00}= & 2P_{I0}-2\frac{\beta_{S}\left(z-1\right)q_{S/00}}{z}P_{00},\\ {\dot{P}}_{\text{S}0}= & P_{SI}+\frac{\beta_{S}\left(z-1\right)q_{S/00}}{z}P_{00}\\ & -\left[\frac{\beta_{S}}{z}+\frac{\beta_{S}\left(z-1\right)q_{S/0S}}{z}+\frac{m_{I}\left(z-1\right)q_{I/S0}}{z}\right]P_{S0},\\ {\dot{P}}_{\text{I}0}= & P_{II}+\frac{m_{I}\left(z-1\right)q_{I/S0}}{z}P_{S0}-\left[\frac{\beta_{S}\left(z-1\right)q_{S/0I}}{z}+1\right]P_{I0},\\ {\dot{P}}_{\text{SS}}= & 2\left[\frac{\beta_{S}}{z}+\frac{\beta_{S}\left(z-1\right)q_{S/0S}}{z}\right]P_{S0}-2\left[\frac{m_{I}\left(z-1\right)q_{I/SS}}{z}\right]P_{SS},\\ {\dot{P}}_{\text{SI}}= & \frac{\beta_{S}\left(z-1\right)q_{S/0I}}{z}P_{0I}+\frac{m_{I}\left(z-1\right)q_{I/SS}}{z}P_{SS}\\ & -\left[\frac{m_{I}}{z}+\frac{m_{I}\left(z-1\right)q_{I/SI}}{z}+1\right]P_{SI},\\ {\dot{P}}_{\text{II}}= & 2\left[\frac{m_{I}}{z}+\frac{m_{I}\left(z-1\right)q_{I/SI}}{z}\right]P_{SI}-2P_{II}. \end{array} \end{array}$$ (3)

In this model, superinfection does not occur because there is only one strain of the pathogen.

#### Mean-field Approximation

To close a set of Eq (3), we approximated several variables by MA, *q*_*σ*_*i*_/*σ*_*j*__ ≈ *ρ*_*σ*_*i*__ (e.g. *P*_S0_ = *ρ*_S_ *q*_0/S_ ≈ *ρ*_S_ *ρ*_0_) and *q*_*σ*_*i*_/*σ*_*j*_*σ*_*k*__ ≈ *ρ*_*σ*_*i*__. In addition, the equations were simplified by the definition of variables from Eq (S1.3) in S1 Appendix.

$$\begin{array}{c} \begin{array}{cc} \dot{\rho_{0}} & =1-\rho_{0}-\rho_{S}\left(1+\beta_{S}\rho_{0}\right),\\ \dot{\rho_{S}} & =\rho_{S}\left(\beta_{S}\rho_{0}-m_{I}\left(1-\rho_{0}-\rho_{S}\right)\right). \end{array} \end{array}$$ (4)

The system has three equilibrium states.

$$\begin{array}{l} {\tilde{E}}_{\text{M}}\;\equiv \;\left({\tilde{\rho }}_{0}^{*},\;{\tilde{\rho }}_{\text{S}}^{*},\;{\tilde{\rho }}_{\text{I}}^{*}\right)=\left(1,\;0,\;0\right),\\ {\hat{E}}_{\text{M}}\equiv \;\left({\hat{\rho }}_{0}^{*},\;{\hat{\rho }}_{\text{S}}^{*},\;{\hat{\rho }}_{\text{I}}^{*}\right)=\left(0,\;1,\;0\right),\\ {\bar{E}}_{\text{M}}\equiv \;\left({\bar{\rho }}_{0}^{*},\;{\bar{\rho }}_{\text{S}}^{*},\;{\bar{\rho }}_{\text{I}}^{*}\right)\\ \;=\;\left(\frac{m_{\text{I}}-1}{\beta_{\text{S}}+m_{\text{I}}},\;\frac{1}{m_{\text{I}}},\;\frac{\beta_{\text{S}}\left(m_{\text{I}}-1\right)}{m_{\text{I}}\left(\beta_{\text{S}}+m_{\text{I}}\right)}\right). \end{array}$$

${\tilde{E}}_{M}$, ${\hat{E}}_{M}$ and ${\bar{E}}_{M}$ mean the states of extinction, disease-free and epidemic, respectively. From local stability analysis of the each equilibrium (see S2 Appendix), ${\tilde{E}}_{M}$ is always unstable, which means that plants do not become extinct at the positive parameter range in the system. When *m*_I_ < 1, ${\hat{E}}_{M}$ is stable, which means that the pathogen is not able to survive when it has a low mortality cost. By contrast, when *m*_I_ exceeds 1, ${\hat{E}}_{M}$ becomes unstable and ${\bar{E}}_{M}$ becomes always stable, which means that the epidemic occurs because of the spread of the pathogens within the plant population. Thus, *m*_I_ = 1 is the threshold value of stability shifting. In conclusion, the stability of the equilibrium states and the equilibrium density of healthy individuals ($\rho_{S}^{*}$) in the epidemic state depend only on *m*_I_, regardless of *β*_S_ in MA.

#### Pair Approximation

To close a set of Eq (3) and consider the effect of local connections on the dynamics, we approximated several variables by PA, $q_{S/0\sigma }\approx q_{S/0}$ and $q_{I/S\sigma }\approx q_{I/S}$. In addition, the equations were simplified by definition of variables (see S1 Appendix), and we obtained following three equilibrium states.

$$\begin{array}{l} {\tilde{E}}_{P}\equiv \left({\tilde{\rho }}_{0}^{*},\;{\tilde{\rho }}_{\text{S}}^{*},\;{\tilde{\rho }}_{\text{I}}^{*},\;{\tilde{q}}_{0/0}^{*},\;{\tilde{q}}_{\text{S}/0}^{*},\;{\tilde{q}}_{\text{I}/0}^{*}\right)\\ \;=\left(1,\;0,\;0,\;1,\;0,\;0\right),\\ {\hat{E}}_{P}\equiv \left({\hat{\rho }}_{0}^{*},\;{\hat{\rho }}_{\text{S}}^{*},\;{\hat{\rho }}_{\text{I}}^{*},\;{\hat{q}}_{0/\text{S}}^{*},\;{\hat{q}}_{\text{S}/\text{S}}^{*},\;{\hat{q}}_{\text{I}/\text{S}}^{*}\right)\\ \;=\left(0,\;1,\;0,\;0,\;1,\;0\right),\\ {\bar{E}}_{P}\;\equiv \left({\bar{\rho }}_{0}^{*},\;{\bar{\rho }}_{\text{S}}^{*},\;{\bar{\rho }}_{\text{I}}^{*},\;{\bar{q}}_{0/0}^{*},\;{\bar{q}}_{\text{S}/0}^{*},\;{\bar{q}}_{\text{I}/0}^{*},\;{\bar{q}}_{0/\text{S}}^{*},\;{\bar{q}}_{\text{S}/\text{S}}^{*},\;{\bar{q}}_{\text{I}/\text{S}}^{*},\;{\bar{q}}_{0/\text{I}}^{*},\;{\bar{q}}_{\text{S}/\text{I}}^{*},\;{\bar{q}}_{\text{I}/\text{I}}^{*}\right)\\ \;=\left(\text{see S}3\text{ Appendix}\right). \end{array}$$

**Fig 1:**
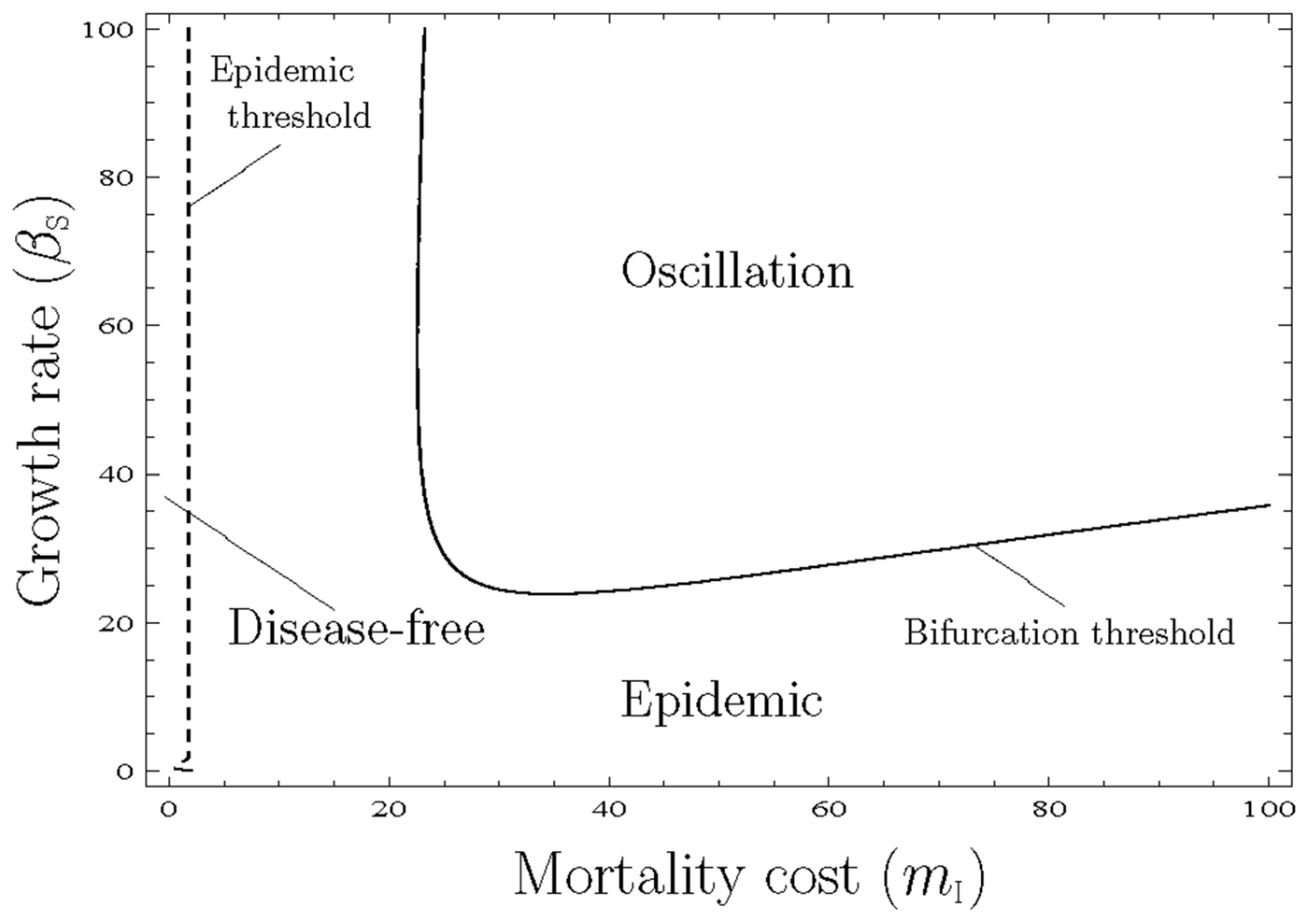
Phase diagram of pair approximation showing the three regions of the equilibrium state. In the epidemic region, plants and pathogens coexist and the equilibrium is stable. In the oscillation region, plants and pathogens coexist, but the equilibrium is unstable. Therefore, Hopf bifurcation occurs and oscillation is observed. The solid line indicates the bifurcation threshold, and the dashed line the epidemic threshold, respectively. In the disease-free region, pathogens become extinct because of the too low mortality cost.

**Fig 2:**
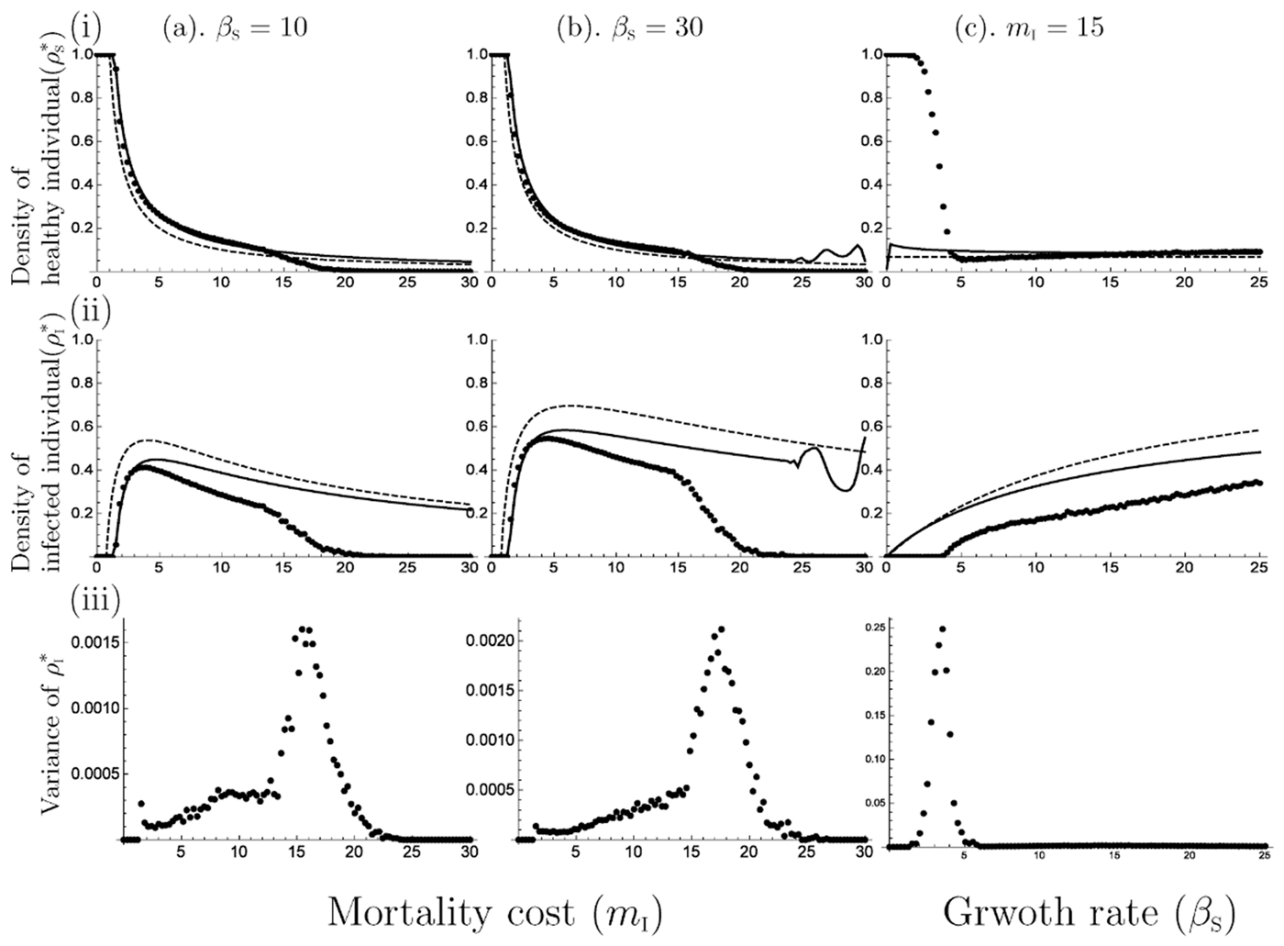
Comparison of the simulation results from mean-field approximation (MA), pair approximation (PA) and Monte Carlo simulation (MCS), depending on mortality cost and growth rate; (a). *β*_S_ = 10, (b). *β*_S_ = 30, (c). *m*_I_ = 15. (i) equilibrium density of healthy individuals ($\rho_{S}^{*}$) (ii) equilibrium density of infected individuals ($\rho_{I}^{*}$). The dots, solid line and dashed line indicate the results of MA, PA and MCS, respectively. These results show that when *m*_I_ is low, both approximation methods present similar trend to MCS, although these methods overestimate the equilibrium value. (iii) The variance among 100 trials in MCS. A high variance means the oscillatory solution is observed.

${\tilde{E}}_{P}$ means that the plants become extinct. Therefore, *q*_*σ*/S_ and *q*_*σ*/I_ are non-existent, because *ρ*_S_ and *ρ*_I_ are equal to 0. ${\hat{E}}_{P}$ means the disease-free state; thus, *q*_*σ*/0_ and *q*_*σ*/I_ are non-existent, because *ρ*_S_ and *ρ*_I_ are non-existent, because *ρ*_S_ and *ρ*_I_ are equal to 0. ${\bar{E}}_{P}$ means the epidemic state, at which state all of the twelve variables exist and have positive values. The local stability of each equilibrium state was examined using the Routh—Hurwitz stability criterion (see S3 Appendix). From the stability analysis, we obtained the three stable-equilibrium phases, disease-free, epidemic, and periodic oscillation (Fig 1). In particular, ${\tilde{E}}_{P}$ is always unstable (i.e. plants do not become extinct), and we derived two thresholds, which are referred to as the epidemic and bifurcation thresholds. The stability of ${\hat{E}}_{P}$ and ${\bar{E}}_{P}$ depends on whether the parameter values exceed each threshold, especially the epidemic condition depends only on the mortality cost, irrespective of the growth rate because the epidemic threshold is ${\left(m_{I}\right)}_{c}=z/\left(z-1\right)$, which is similar to MA. That is, if *m*_I_ is low, pathogens become extinct (panels (a) and (b) in Fig 2), because the low *m*_I_ means a high virulence or low infection rate, thus pathogens die within the infected hosts before infecting other hosts. In addition, a large *m*_I_ leads to a decrease in both healthy and infected individuals. *β*_S_ affects the equilibrium value in the epidemic phase. For example, a large *β*_S_ leads to an increase in the equilibrium density of infected individuals (panel (c) in Fig 2), because pathogens can spread widely within the many hosts supplied by the fast growth rate. In the epidemic phase, when *β*_S_ is large, the equilibrium density of healthy individuals ($\rho_{S}^{*}$) decreases, thus a low growth rate has an advantage over a high growth rate for plants in this phase. In addition, Hopf bifurcation occurs when the parameter values exceed to the bifurcation threshold (panel (b) in Fig 2). In other words, the stability of ${\hat{E}}_{P}$ shifts from stable to unstable, which is different from MA.

To check the validity of each approximation method, we compared equilibrium values among MA, PA and MCS. The MCS was conducted 100 times for each given parameter set; (a). *β*_S_ = 10 and *m*_I_ = 0 ∼ 30, (b). *β*_S_ = 30 and *m*_I_ = 0 ∼ 30, (c). *m*_I_ = 15 and *β*_S_ = 0 ∼ 25 in Fig 2. We used a two-dimensional square lattice torus and calculated an average value of 100 trials at each parameter value (Fig 2). The result indicated that there are several discrepancies with respect to the equilibrium values and threshold values among them: MA and PA overestimate the equilibrium values and the periodic solution is observed in PA under higher parameter values compared with MCS. In Fig 2, the discrepancy of the threshold value between MCS and MA (PA) is large when the mortality cost is large ((*a*), (*b*)) or the growth rate is low ((*c*)). The use of these approximation methods, in which we neglected the effect from far sites, is probably the cause of the discrepancies. However, the periodic solution was observed in the result of PA and MCS [35], which was differently from MA. Thus, PA could explain the basic behavior of the system better than MA.

### Multiple-strain models

We examined four models (*n* = 3, 4, 11, and 26) in GM (Eq (2)); however, the analysis was too complex to obtain the analytical result because of too many variables (e.g. MA and PA require at least *n* and $\Sigma_{i=1}^{n}i-1$ variables, respectively). Therefore, we derived the equilibrium value by MA in the 2-strain and 3-strain models, and analyzed all the models through computer simulations (MCS and numerical simulation of MA and PA). We set *m*_I_ = *m*_*i*−1_+*Δm*_*i*,*i*−1_(*i* = 2, 3,…, *n*−1), *s* = 0, 0.5, 1.0, 1.5 and vary the *β*_S_ in the simulations. In this paper, we assumed that *Δm*: = *Δm*_*i*,*i*−1_ was constant just for the simplicity of the model. As a result of the numerical simulations of MA and PA, the healthy individuals did not become extinct in all models. However, the healthy and infected individuals did become extinct in MCS, especially when *n* is large and *β*_S_ is small. From the comparison among MA, PA and MCS, discrepancies became large as the number of strains increased.

#### The 2-strain model (*n* = 3)

We obtained five equilibrium states by MA (Table 1 in detail): ***E***_1_: extinction ($\rho_{0}^{*}=1$), ***E***_2_: disease-free ($\rho_{I_{1}}^{*}=0$, $\rho_{I_{2}}^{*}=0$), ***E***_3_: occupation of a strain with high cost ($\rho_{I_{1}}^{*}=0$, $\rho_{I_{2}}^{*}>0$), ***E***_4_: occupation of a strain with low cost ($\rho_{I_{1}}^{*}>0$, $\rho_{I_{2}}^{*}=0$), ***E***_5_: coexistence ($\rho_{I_{1}}^{*}>0$, $\rho_{I_{2}}^{*}>0$). In particular, in the (equilibrium) phase of coexistence (i.e. ***E***_5_ is stable), the dominant strain changes depending on the plant growth rate.

**Table 1:** Equilibria of the 2-strain model in MA.

***E***_1_	(1, 0, 0, 0)
***E***_2_	(0, 1, 0, 0)
***E***_3_	$\left(\frac{m_{2}-1}{\beta_{S}+m_{2}},\;\frac{1}{m_{2}},\;0,\;\frac{\beta_{S}(m_{2}-1)}{m_{2}(\beta_{S}+m_{2})}\right)$
***E***_4_	$\left(\frac{m_{1}-1}{\beta_{S}+m_{1}},\;\frac{1}{m_{1}},\;\frac{\beta_{S}(m_{1}-1)}{m_{1}(\beta_{S}+m_{1})},\;0\right)$
***E***_5_	$\left(\frac{m_{2}-m_{1}}{s\beta_{S}m_{1}},\;\frac{m_{1}(s\beta_{S}+1)-m_{2}}{\beta_{S}(m_{2}+m_{1}(s-1))},\;\frac{s\beta_{S}m_{1}(m_{2}-1)-(m_{2}-m_{1})(\beta_{S}+m_{2})}{s\beta_{S}m_{1}(m_{2}+m_{1}(s-1))},\;\frac{m_{1}(m_{2}-m_{1})+\beta_{S}(m_{2}-m_{1}-sm_{1}(m_{1}-1))}{s\beta_{S}m_{1}(sm_{1}+m_{2}-m_{1})}\right)$

$E\equiv (\rho_{0}^{*},\;\rho_{S}^{*},\;\rho_{I_{1}}^{*},\;\rho_{I_{2}}^{*})$.

**Fig 3:**
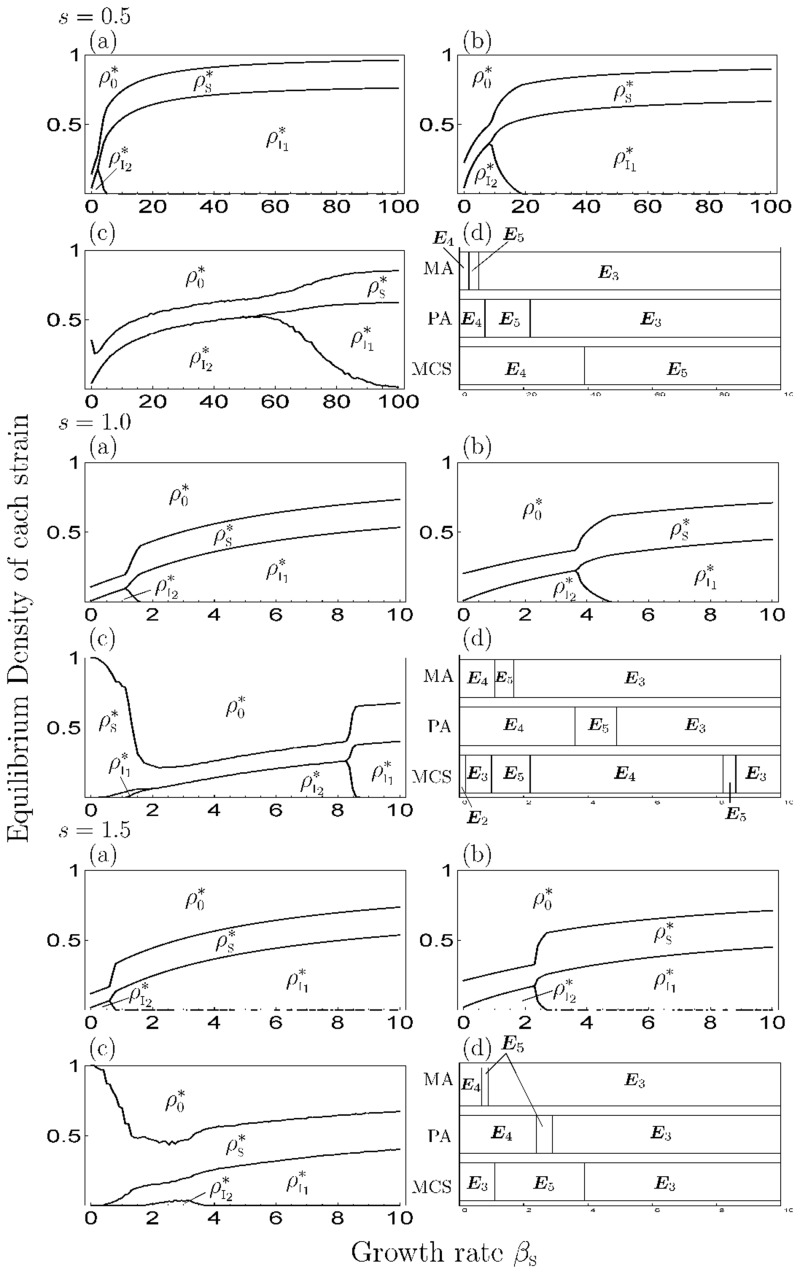
The equilibrium value of each state and transition of the equilibrium phase depending on the superinfection rate in the 2-strain model. We set *m*_1_ = 5 and *Δm* = 5. The I, II and III differ in the value of *s* (=0.5, 1.0, 1.5). (a)–(c) show the variation of the equilibrium density of each state (“0”, “S”, “I_1_” and “I_2_”. *Σ*_*σ*_ *ρ*_*σ*_ = 1) with growth rate in each simulation: (a) MA, (b) PA, (c) MCS. (d) shows the transition of the equilibrium phase with *β*_S_ in MA, PA, and MCS.

**Fig 4:**
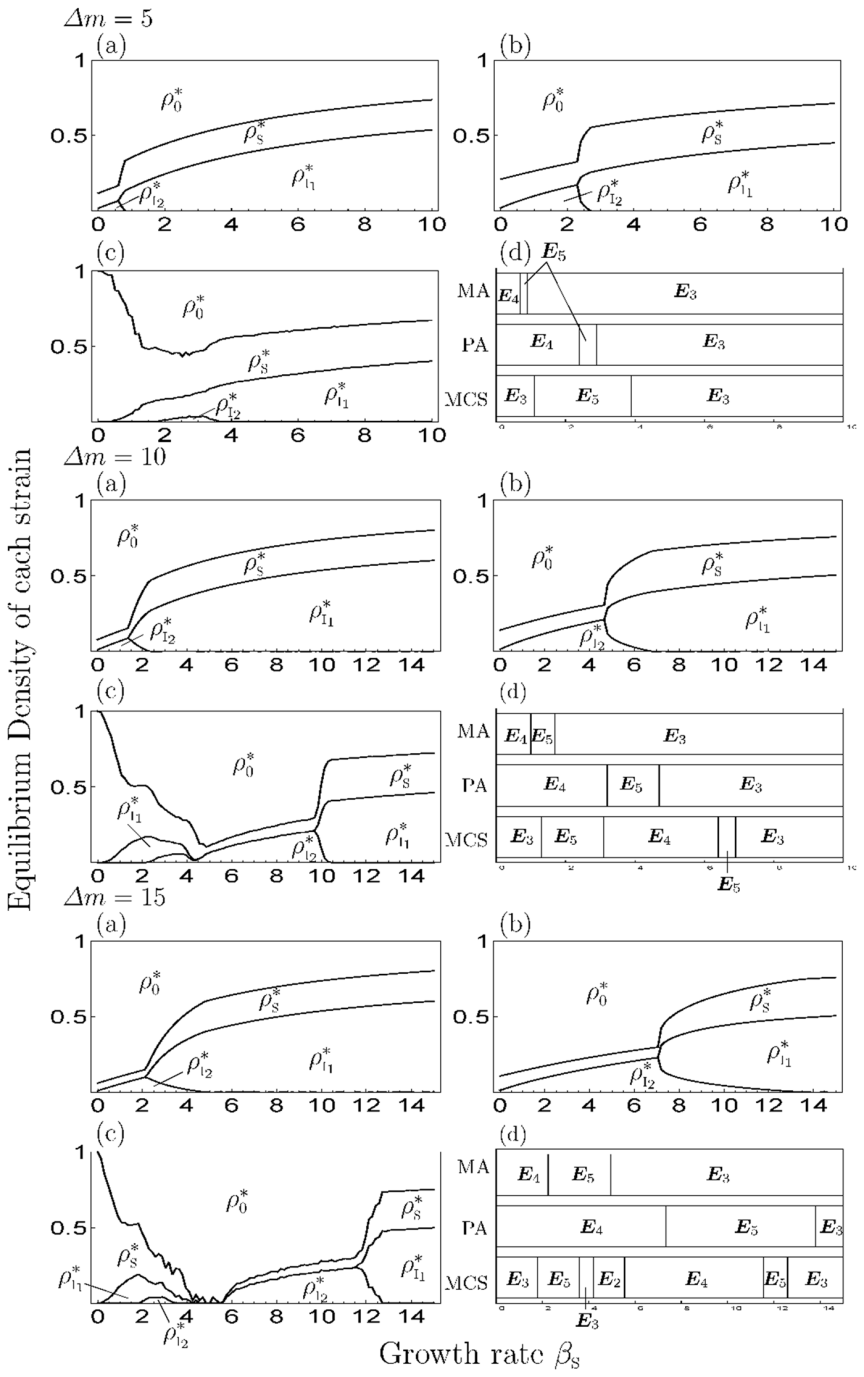
The equilibrium value of each strain and transition of the equilibrium phase depending on the difference of mortality cost in the 2-strain model. We set *m*_I_1__ = 5 and *Δm* = 5 in all figures. The I, II and III differ in the value of *s* (=0.5, 1.0, 1.5). (a) MA, (b) PA, (c) MCS, (d) the transition of equilibrium phase.

When *s* > 0, Figs 3 and 4 show the equilibrium density of each state depending on the growth rate (*β*_S_) for a given superinfection rate (*s*) and the difference in mortality cost (*Δm*), respectively, in the simulation of MA (panels (a)), PA (panels (b)) and MCS (panels (c)) and transition of equilibrium phase (panels (d)). As a result, when *β*_S_ is small, the strain with a high cost is dominant, then the dominant strain shifts to strain with a low cost as *β*_S_ increases and the strain occupies the pathogen population on a large *β*_S_. The increase in *s* and decrease in *Δm* lead to lower threshold values of the phase transition (panels (d) in Figs 3 and 4); thus, when *s* is small or *Δm* is large, the parameter range of the phase of coexistence increases. Notably, when *Δm* is large (moderate competition), multiple strains are more likely to coexist, like ‘limiting similarity of niche’ proposed by competition theory [39, 40]. For the healthy individuals, they increase their density at the coexistence phase and decrease it at other phases in PA. Thus, if the growth rate is out of the range value of the coexistence phase, healthy individuals do not increase their population.

**Fig 5:**
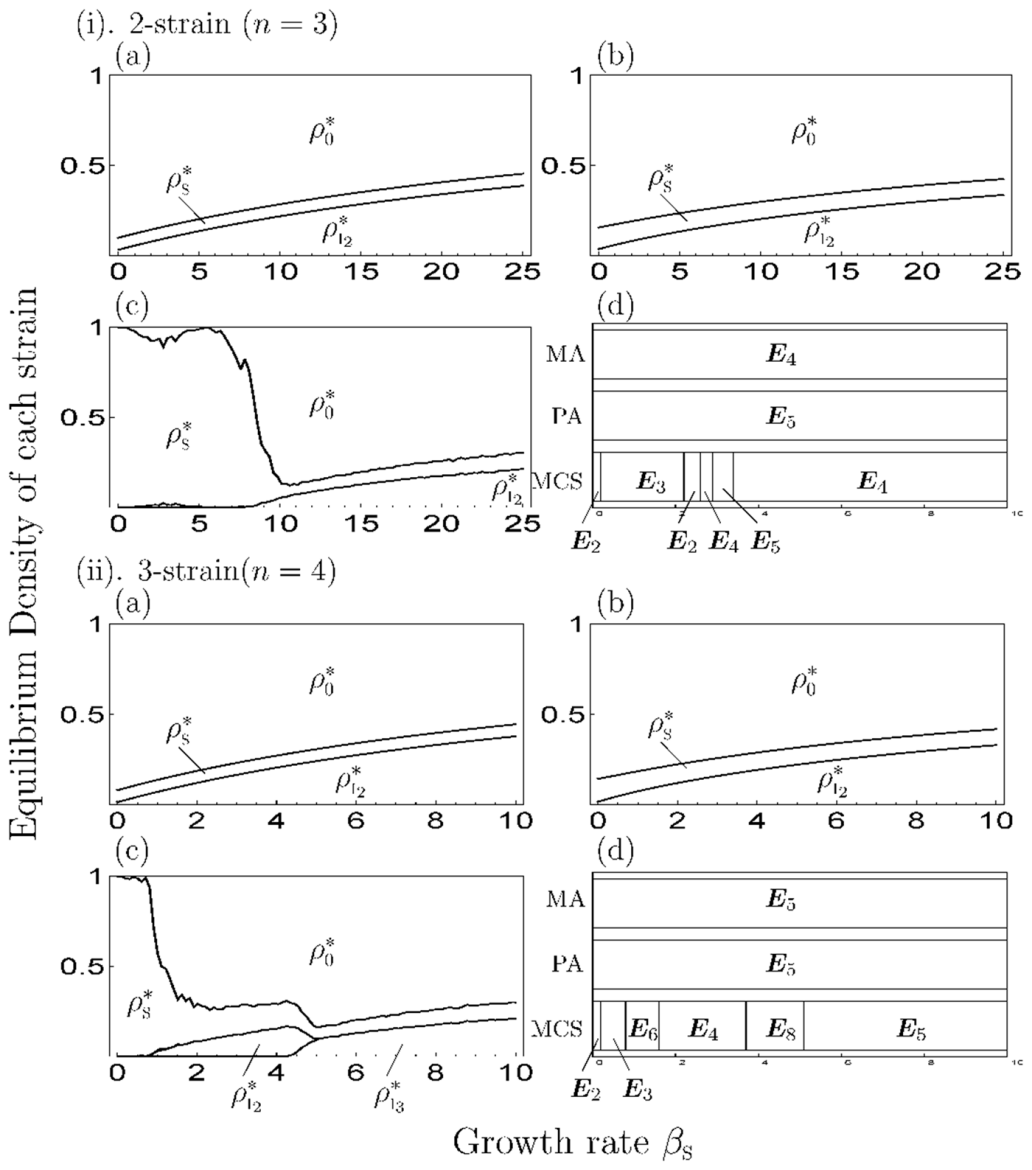
The case of no superinfection (*s* = 0) in the 2-strain and 3-strain model by MA, PA and MCS. We plotted the global density of each strain of the pathogen at the equilibrium state depending on *β*_S_. The black circles and triangles indicate I_1_ and I_2_, respectively, and squares indicate I_3_ in (b). The parameter values are (a) *n* = 3, *m*_I_1__ = 5, *Δm* = 10, (b) *n* = 4, *m*_I_1__ = 5, *Δm* = 5.

When *s* = 0 (no superinfection), the shift of the equilibrium phase along the gradient of *β*_S_ is different from the case of *s* > 0 in MCS. For example, when *β*_S_ is large, the strain with the highest mortality cost occupies and coexists with healthy plants (i.e. strains with lower cost become extinct). In addition, the range of the coexistence phase decreases compared with the case of when *s* > 0 (Fig 5(i)). In MA and PA, the strain with a high cost always occupies regardless of other parameter values (panels (a) and (b) in Fig 5(i)). Thus, when *s* = 0 and *β*_S_ is large, the pathogen population is occupied by the strain with highest cost, contrary to the case of *s* > 0.

**Fig 6:**
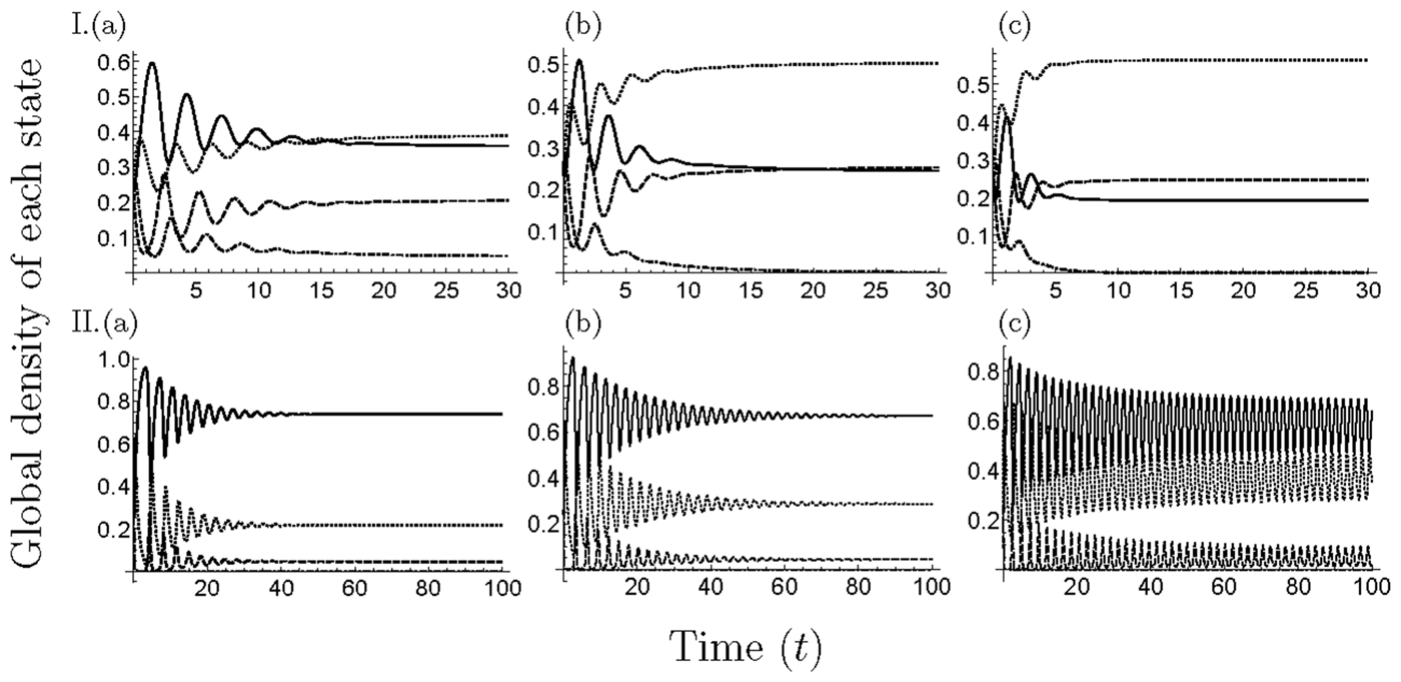
Time development in the 2-strain model. We plotted the equilibrium value of the global density of each state. In all figures, we set *s* = 1.0 and *Δm* = 15. I. *m*_I_1__ = 5, II. *m*_I_1__ = 30 and (a) *β*_S_ = 10, (b) *β*_S_ = 15, (c) *β*_S_ = 25.

From the comparison of numerical simulations (MA and PA) with MCS, when *β*_S_ is small, the result is at extreme variance with MCS. Thus, in the parameter range, the approximation method cannot apply. When *β*_S_ is large enough, the discrepancy between them increases with the decrease in *s* (Fig 3) and *Δm* (Fig 4). However, MA and PA can explain the transition process of the equilibrium phase (panels (d) in Figs 3 and 4). In addition, the oscillatory solution, which means the solution is oscillating for a long time although unproven, is not observed in the coexistence phase (Fig 6). However, in the occupation phase of a strain, the oscillatory solution is observed because the behavior of the model follows the 1-strain model in that phase.

#### The 3-strain model (*n* = 4)

We obtained nine equilibrium states by MA (Table 2 in detail): ***E***_1_: extinction, ***E***_2_: disease-free, ***E***_3−5_: occupation of a strain ($\rho_{I_{i}}^{*}>0$, $\rho_{I_{j}}^{*}=0$, $\rho_{I_{k}}^{*}=0$), ***E***_6−8_: coexistence of two strains ($\rho_{I_{i}}^{*}>0$, $\rho_{I_{j}}^{*}>0$, $\rho_{I_{k}}^{*}=0$), ***E***_9_: coexistence of all strains ($\rho_{I_{i}}^{*}>0$, $\rho_{I_{j}}^{*}>0$, $\rho_{I_{k}}^{*}>0$).

**Table 2:** Equilibria of the 3-strain model in MA.

***E***_1_	(1, 0, 0, 0, 0)
***E***_2_	(0, 1, 0, 0, 0)
***E***_3_	$\left(\frac{m_{1}-1}{\beta_{S}+m_{1}},\;\frac{1}{m_{1}},\;\frac{\beta_{S}(m_{1}-1)}{m_{1}(\beta_{S}+m_{1})},\;0,\;0\right)$
***E***_4_	$\left(\frac{m_{2}-1}{\beta_{S}+m_{2}},\;\frac{1}{m_{2}},\;0,\;\frac{\beta_{S}(m_{2}-1)}{m_{2}(\beta_{S}+m_{2})},\;0\right)$
***E***_5_	$\left(\frac{m_{3}-1}{\beta_{S}+m_{3}},\;\frac{1}{m_{3}},\;0,\;0,\;\frac{\beta_{S}(m_{3}-1)}{m_{3}(\beta_{S}+m_{3})}\right)$
***E***_6_	$\left(\frac{m_{2}-m_{1}}{s\beta_{S}m_{1}},\;\frac{m_{1}(s\beta_{S}+1)-m_{2}}{\beta_{S}(m_{2}+m_{1}(s-1))},\;\frac{s\beta_{S}m_{1}(m_{2}-1)-(m_{2}-m_{1})(\beta_{S}+m_{2})}{s\beta_{S}m_{1}(m_{2}+m_{1}(s-1))},\;\frac{m_{1}(m_{2}-m_{1})+\beta_{S}(m_{2}-m_{1}-sm_{1}(m_{1}-1))}{s\beta_{S}m_{1}(sm_{1}+m_{2}-m_{1})},\;0\right)$
***E***_7_	$\left(\frac{m_{3}-m_{1}}{s\beta_{S}m_{1}},\;\frac{m_{1}(s\beta_{S}+1)-m_{3}}{\beta_{S}(m_{3}+m_{1}(s-1))},\;\frac{s\beta_{S}m_{1}(m_{3}-1)-(m_{3}-m_{1})(\beta_{S}+m_{3})}{s\beta_{S}m_{1}(m_{3}+m_{1}(s-1))},\;0,\;\frac{m_{1}(m_{3}-m_{1})+\beta_{S}(m_{3}-m_{1}-sm_{1}(m_{1}-1))}{s\beta_{S}m_{1}(sm_{1}+m_{3}-m_{1})}\right)$
***E***_8_	$\left(\frac{m_{3}-m_{2}}{s\beta_{S}m_{2}},\;\frac{m_{2}(s\beta_{S}+1)-m_{3}}{\beta_{S}(m_{3}+m_{2}(s-1))},\;0,\;\frac{s\beta_{S}m_{2}(m_{3}-1)-(m_{3}-m_{2})(\beta_{S}+m_{3})}{s\beta_{S}m_{2}(m_{3}+m_{2}(s-1))},\;\frac{m_{2}(m_{3}-m_{2})+\beta_{S}(m_{3}-m_{2}-sm_{2}(m_{2}-1))}{s\beta_{S}m_{2}(sm_{2}+m_{3}-m_{2})}\right)$
***E***_9_	$\begin{array}{l} (\frac{m_{1}m_{3}-m_{2}}{\beta_{S}m_{2}+m_{1}m_{3}},\;\frac{m_{2}}{m_{1}m_{3}},\;\frac{\beta_{S}m_{2}\left(s\left(m_{1}m_{3}-m_{2}\right)-m_{3}+m_{2}\right)-m_{1}m_{3}\left(m_{3}-m_{2}\right)}{sm_{1}m_{3}\left(\beta_{S}m_{2}+m_{1}m_{3}\right)},\\ \frac{m_{2}\left(m_{3}-m_{1}\right)-sm_{1}\left(m_{2}+m_{1}m_{3}\right)}{sm_{1}m_{3}\left(\beta_{S}m_{2}+m_{1}m_{3}\right)},\;\frac{m_{1}m_{3}\left(s\beta_{S}m_{1}-m_{2}+m_{1}\right)-\beta_{S}m_{2}\left(sm_{1}+m_{2}-m_{1}\right)}{sm_{1}m_{3}\left(\beta_{S}m_{2}+m_{1}m_{3}\right)}) \end{array}$

$E\equiv (\rho_{0}^{*},\;\rho_{S}^{*},\;\rho_{I_{1}}^{*},\;\rho_{I_{2}}^{*},\;\rho_{I_{3}}^{*})$.

**Fig 7:**
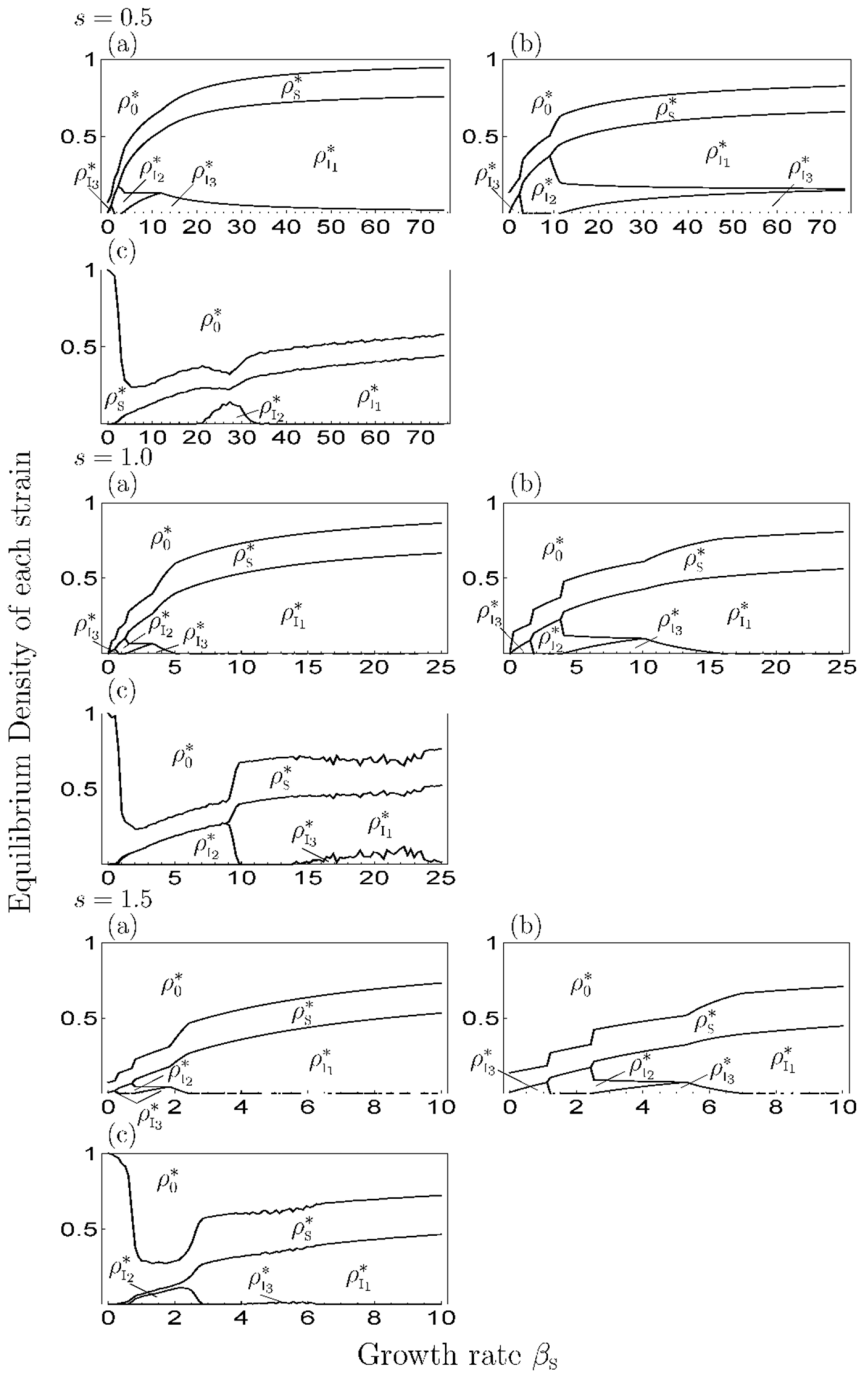
The equilibrium value of each strain depending on the superinfection rate in the 3-strain model. We set *m*_I_1__ = 5 and *Δm* = 5 in all figures. The I, II and III differ in the value of *s* (=0.5, 1.0, 1.5). (a) MA, (b) PA, (c) MCS.

**Fig 8:**
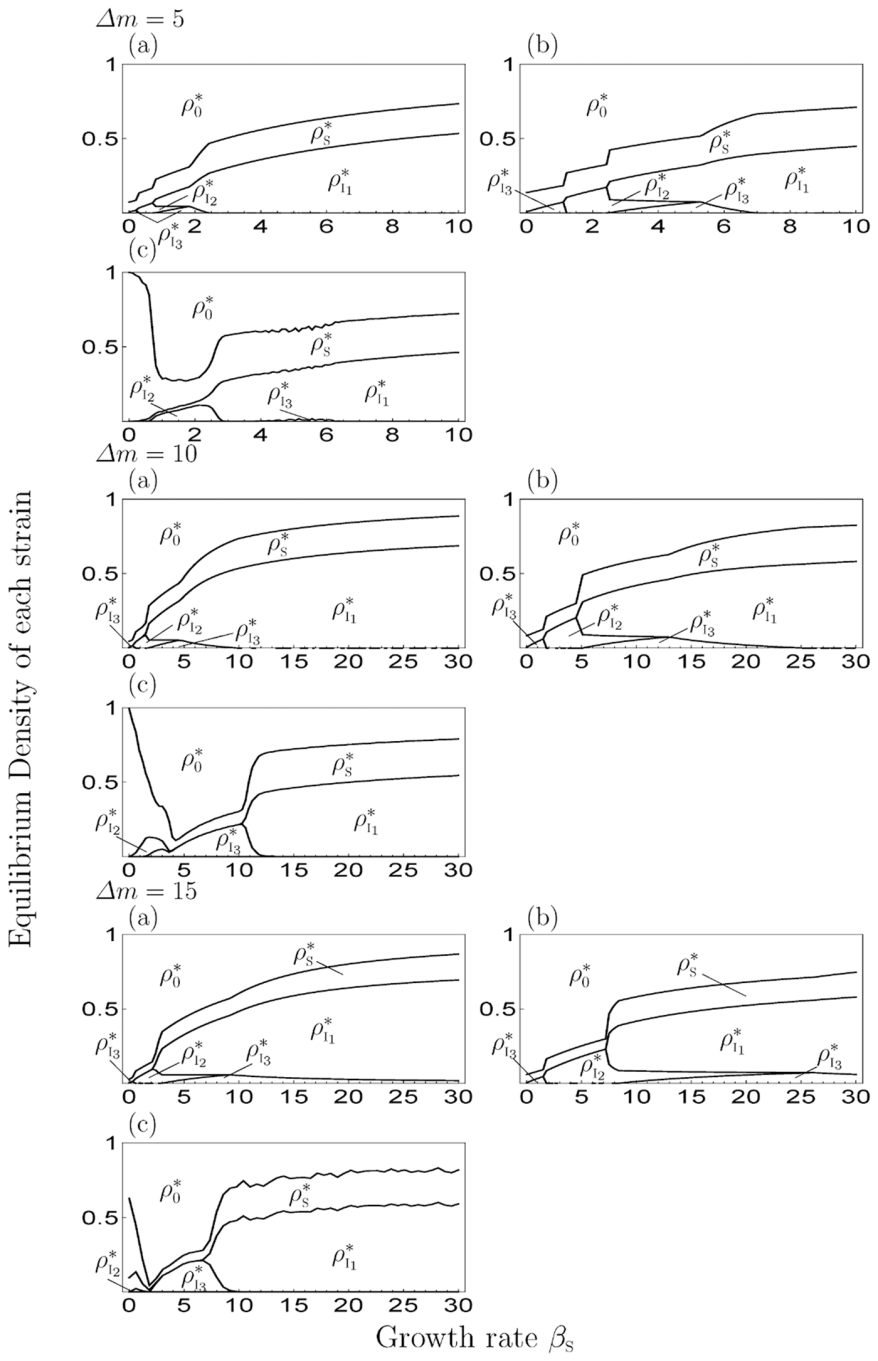
The equilibrium value of each strain depending on the difference in the mortality cost in the 3-strain model. We set *m*_I_1__ = 5 and *s* = 1.5 in all figures. The I, II and III differ in the value of *Δm* (=5, 10, 15). (a) MA, (b) PA, (c) MCS.

**Fig 9:**
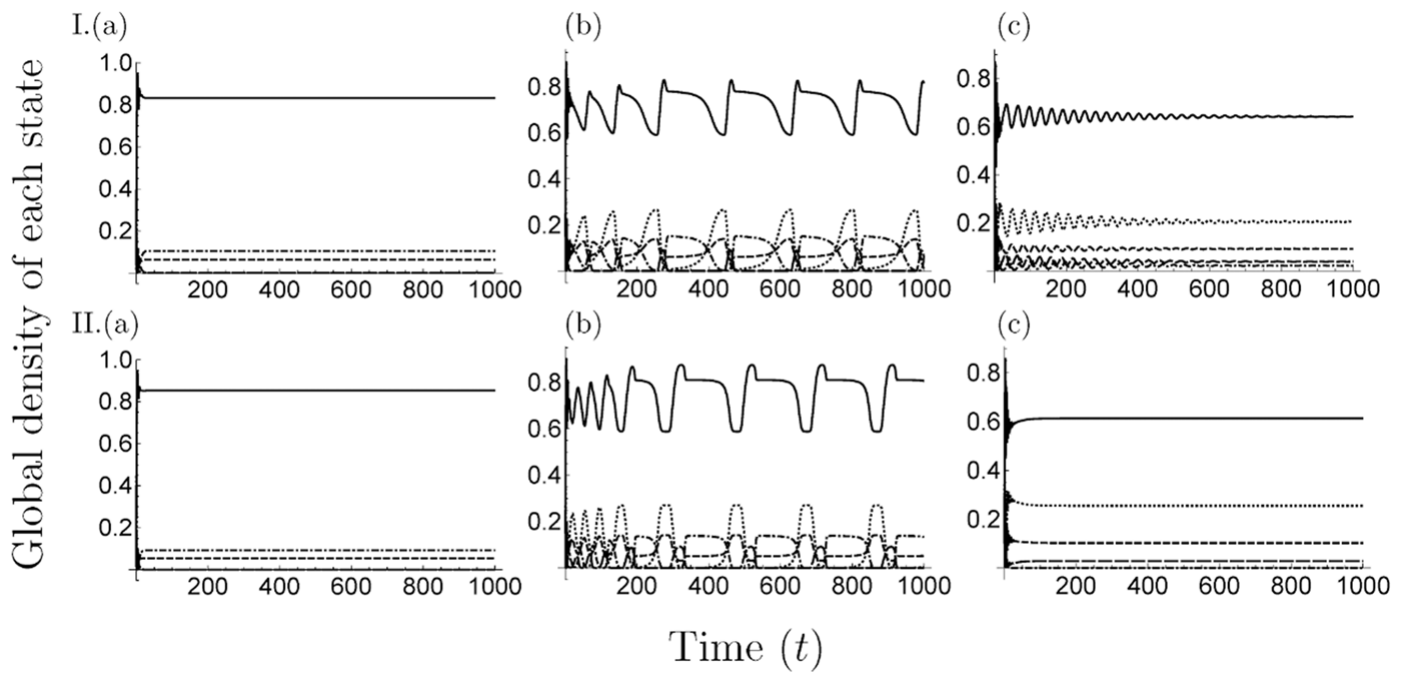
Time development of global density of each state in the 3-strain model. We plotted the equilibrium value of the global density of each state. We set the parameter values; I. *s* = 1.0, *m*_I_1__ = 10, *Δm* = 15, II. *s* = 1.5, *m*_I_1__ = 10, *Δm* = 20 and (a) *β*_S_ = 3, (b) *β*_S_ = 5, (c) *β*_S_ = 7. In the 3-strain model, the oscillatory solution was observed in a particular parameter range at the coexistence phase (panel(b)).

When *s* > 0, Figs 7 and 8 show the equilibrium density of each state depending on *β*_S_ in a given *s* and the difference in mortality costs (*Δm*), respectively, by the numerical simulation of MA (panels (a)), PA (panels (b)) and MCS (panels (c)). Among them, the occupation phase of a strain and the coexistence phase of two strains showed a generally similar response to the parameter values in the 1-strain and 2-strain models. The effect of *s* and *Δm* on equilibrium values, threshold values and discrepancies among simulation results were similar to the 2-strain model. Thus, the increase or decrease of the density of healthy individuals depends on *β*_S_ in PA, which is different from MA (i.e. the growth rate negatively affects the density of healthy individuals in MA). However, when *s* and *Δm* are both large, the transition of the equilibrium phase in MA (PA) was different from the MCS, because the strain with highest cost (I_3_) became extinct in the MCS. In addition, the oscillatory solution was observed in a parameter range (Fig 9) and the strain with the middle cost (I_2_) was dominant, similar to the results of the previous studies [17–21](Figs 7 and 8). In addition, when *s* = 0 (Fig 5(ii)), the response to *β*_S_ was the same as in the 2-strain model (i.e. the range of the coexistence phase decreased and pathogen population was occupied by strain with the highest cost at a large *β*_S_).

#### Multiple-strain models

In the multiple-strain models, there are many equilibrium states: extinction, disease-free, occupation of a strain, coexistence of various strains, and coexistence of all strains.

**Fig 10:**
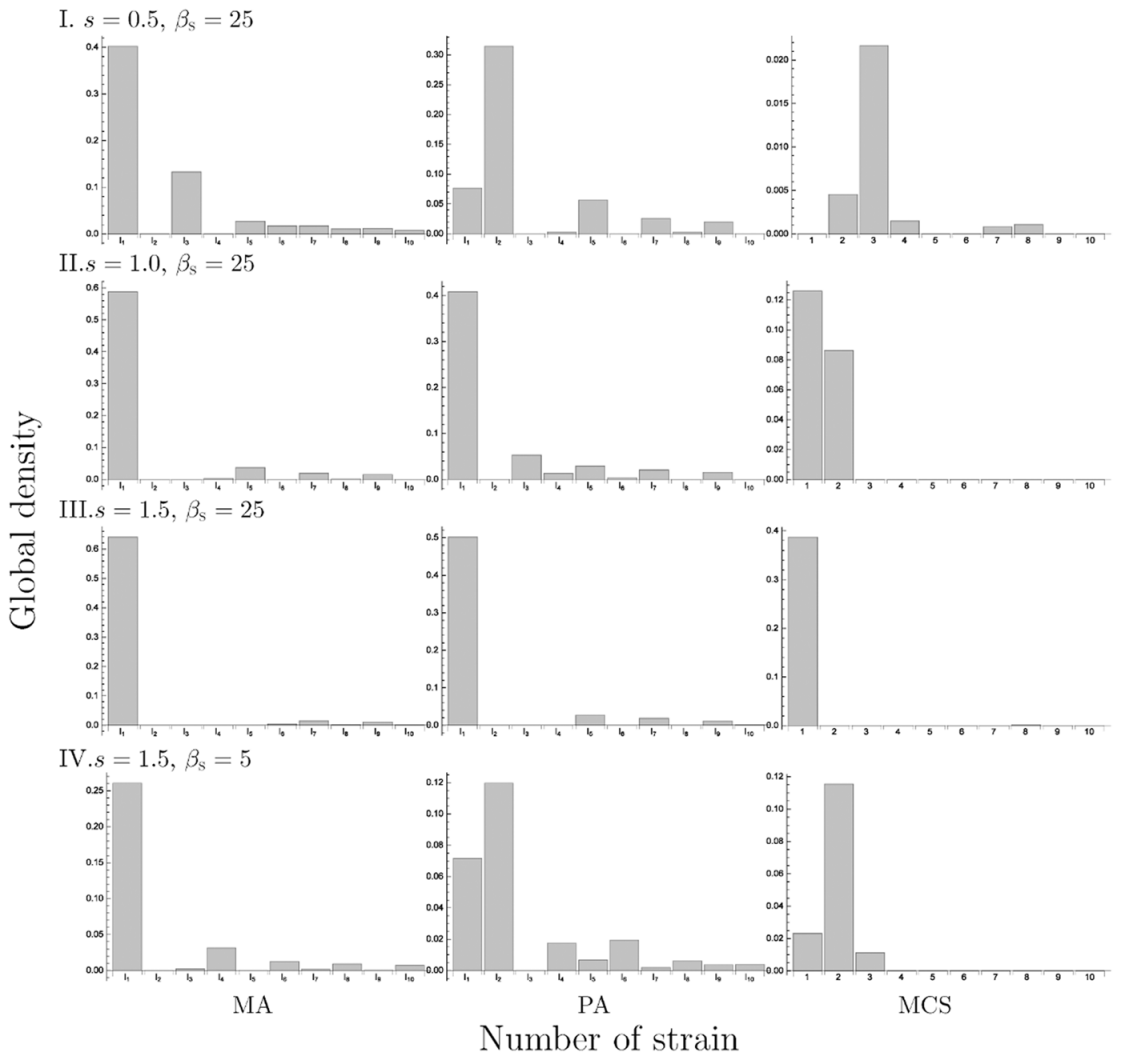
The equilibrium density distribution of strains in 10-strain model. The left, center and right panels show the result in simulation by MA, PA and MCS, respectively. We set *m*_I_1__ = 5, *Δm* = 5 and other parameter values are: I. *s* = 0.5, *β*_S_ = 25, II. *s* = 1.0, *β*_S_ = 25, III. *s* = 1.5, *β*_S_ = 25, IV. *s* = 1.5, *β*_S_ = 5.

**Fig 11:**
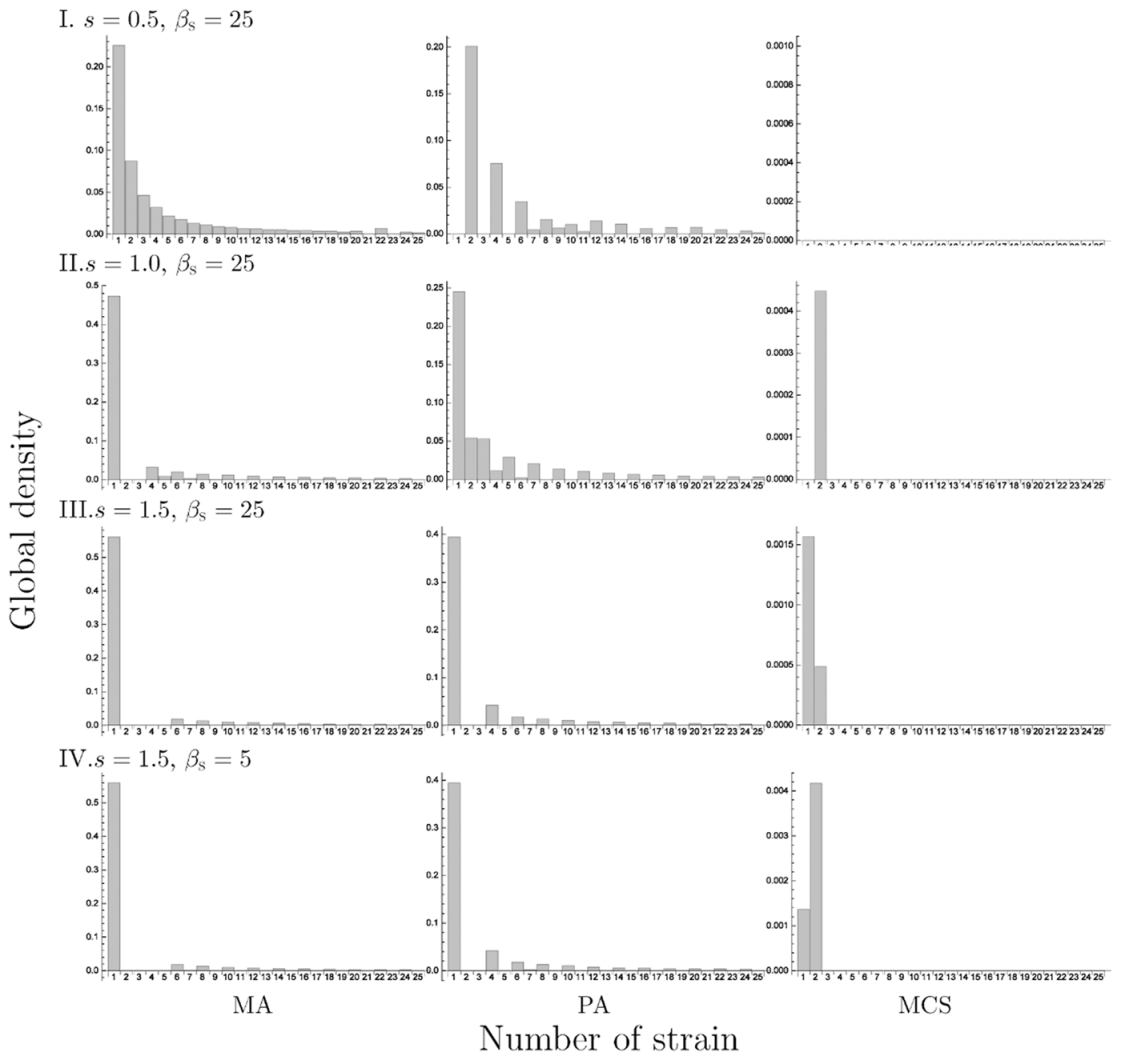
The equilibrium density distribution of strains in the 25-strain model. The left, center and right panels show the result in simulation by MA, PA and MCS, respectively. We set *m*_I_1__ = 5, *Δm* = 5 and other parameter values are: I. *s* = 0.5, *β*_S_ = 5, II. *s* = 0.5, *β*_S_ = 25, III. *s* = 1.0, *β*_S_ = 25, IV. *s* = 1.5, *β*_S_ = 25.

**Fig 12:**
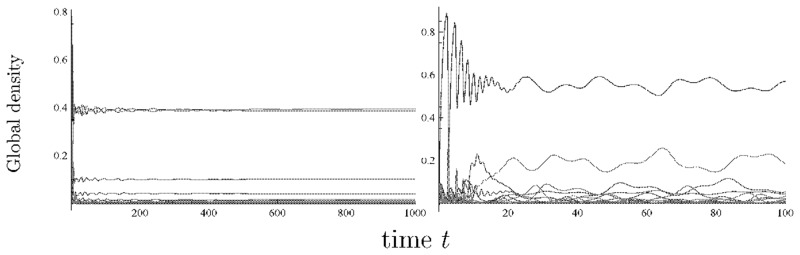
Time development in the 25-strain model. We plotted the equilibrium value of the global density of each state. In the all figures, we set *m*_I_1__ = 5, *Δm* = 5 and *β*_S_ = 5, and (a) *s* = 1.5, (b) *s* = 0.5.

We plotted the results of the 10-strain (Fig 10) and 25-strain models (Fig 11) at *Δm* = 5 with varying *β*_S_ and *s*. As a result, a smaller value of *β*_S_ or *s* led to the dominance of the strain with higher cost (Figs 10 and 11) and the cost of the dominant strain shifted to a lower value as these parameter values increased, similar to the 2-strain and 3-strain model. In addition, when the *β*_S_ was small, the oscillatory solution was observed (e.g. Fig 12), and when the *s* was also small, there was a possibility of extinction in MCS when the *n* was too large (Fig 11).

## Discussion

The physical connection through ramets, especially through the vascular system of plants, is important to spread pathogens within a clonal plant population [25]. Viruses are able to spread through the vascular system [41], and fungal pathogens are able to grow their hyphae through the intercellular spaces of vascular vessels [42]. By contrast, plants have evolved to increase the clonal growth rate to escape from disease when (systemic) pathogens invade the population [8, 9, 26–28]. Thus, clonal plants and pathogens have been mutually affected in the course of evolution.

Several studies have analyzed the pathogen spread process by an approximation method on the lattice space [34–36] and the superinfection process using mathematical models without spatial structures [18]. Sato et al. (1994) studied the case of *d*_s_ = 1 in our 1-strain model. Their model had three equilibrium states, (disease-free, endemic and epidemic), and they derived equilibrium values at two states (disease-free and endemic) and their local stability condition, explicitly. However, they did not obtain the epidemic equilibrium value and its local stability. Haraguchi and Sasaki (2000) considered that there is no trade-off between the infection rate and the virulence of a pathogen and assumed that multiple pathogens have different virulences. They examined the ESS of the infection rate by numerical simulation and discussed the evolution of the infection rate of the pathogen. Their simulation suggested that pathogens evolve to an intermediate infection rate. Satulovsky and Tomé (1994) considered a transition rule similar to our model and assumed the correlation between the transition rates of each state (i.e. *β*_S_ + *m*_I_ + *d*_I_ = 1). According to their results of PA and MCS, there are four equilibrium phases (three stationary and one oscillation phase), and if the transition rate to state 0 (*d*_I_) is small, the Hopf bifurcation occurs. Nowak and May (1994) examined the superinfection events using ODE (Ordinary Differential Equation). As a result, superinfection leads to maintenance of the polymorphism of a parasite strain, and the oscillatory solution (i.e. competition among plants and pathogens) is observed when there is more than one strains. However, they did not consider the spatial structure and host reproductive dynamics (they assumed that the host increases constantly).

To consider the effect of spatial structures, we analyzed plant reproduction and pathogen propagation dynamics with superinfection in a clonal plant population on the lattice space. We analyzed five models (the 1-strain model and four multiple-strain models), including interaction among plant and several strains of pathogen, and adopted the MA and PA to analyze the dynamics of the models. In addition, we checked the validity of the approximation methods in comparison with MCS.

Using the 1-strain model (*n* = 2), the value of the epidemic threshold depended only on the mortality cost, which means that to establish a pathogen within a plant population depends only on the ability of the pathogens, regardless of plants. In addition, the density of healthy individuals decreases with increasing growth rate and mortality cost (panels (i) in Fig 2). By contrast, the density of infected individuals increases with the growth rate and decreases with the increase in mortality cost (panels (ii) in Fig 2). Therefore, plants should not increase their growth rate to maintain a large population size when infected by a systemic pathogen, and the pathogen should evolve a low mortality cost that is higher than the epidemic threshold to maintain their population and that of their hosts.

In addition, if the parameter values exceed the bifurcation threshold, the Hopf bifurcation occurs and the periodic solution, which means plants and pathogens continue to compete forever, is observed in PA and MCS, which is different from MA. Thus, considering the effect of local interaction is important to express the dynamics of the pathogen propagation process. However, MA (which neglect the local interaction) is useful to analyze the dynamics in a measure such as the number of equilibrium states in the model.

In the multiple-strain models (*n* > 2), we assumed that multiple strain of a pathogen had different mortality costs, and that the already-infected individuals are superinfected by strains with lower mortality costs. The analytical result of MA showed that there are a lot of equilibrium states: extinction, disease-free, occupation of a strain, coexistence of various strains, and coexistence of all strains (Tables 1 and 2). From the results of the simulation of MA, PA and MCS, the equilibrium phase and the dominance of a strain in the coexistence phases depend on parameter values and the oscillatory solution is observed in the coexistence phases, except for the 2-strain model in PA and MCS (Figs 6 and 9).

In addition, the genetic diversity of a pathogen is maintained by a decrease in superinfection events. In fact, the parameter range of the coexistence phase increases with the decrease in superinfection rates, even when the difference in mortality cost is small. This is because a superinfection event is conducive to the strong competition among strains from a decrease in the difference of spread-speed among them by additional transmission routes. But, if superinfection does not occur (*s* = 0), the range of the coexistence phase decreases (Fig 5), thus superinfection is important to maintain genetic diversity. However, when the plant growth rate increases, the pathogen population is occupied by a strain eventually, regardless of the superinfection rate. (Figs 3, 5 and 7). That is, the increase in the growth rate causes a decrease in the genetic diversity of pathogen. For healthy individuals, too high a growth rate provides them with no benefit. Healthy individuals can increase their abundance via the growth rate in the coexistence phase of several strains; however, if the growth rate is too high, an equilibrium phase shifts to the phase of occupation of one strain, and the density of healthy individuals then decreases with increasing growth rate. The dynamics of the model follows the 1-strain model in this phase.

In summary, pathogens maintain their genetic diversity through superinfection events and a moderate mortality cost relative to growth rate; thus they evolve their mortality cost and superinfection rate depending on plant growth ability to maintain their population. By contrast, the number of healthy individuals (plants) increases in (all and several strains) coexistence phases with the growth rate. Thus, when systemic pathogens invade the plant population, plants evolve the growth rate to be slightly lower than a threshold value at which the equilibrium phase shifts to the phase of occupation of a strain to increase their population.

In this paper, we constructed simple models to express the relationship between clonal growth and pathogen spread through superinfection, considering the spatial structure. We showed that: (i) The strain with an intermediate cost becomes dominant, similar to the previous studies [17–21], when both the superinfection rate and the growth rate are low. However, a high superinfection rate or growth rate leads to dominance of the strain with lowest cost in our model. Actually, pathogen gets more benefit due to low mortality cost when hosts grow rapidly [3]. (ii) The competition among strains occurs in the coexistence phase of various strains by PA and MCS in the model of *n* > 3. (iii) Too high a growth rate leads to occupation of the strain with lowest cost. Thus, the competition between the strain and the hosts occurs, thereby the host population decreases in all models. (iv) Pathogens easily maintain their genetic diversity with a low superinfection rate. However, if they do not superinfect, such maintenance becomes difficult. (v) When the growth rate of a plant is low, an individual at a local site is strongly interconnected by distant individuals because MA and PA do not apply in this case. In conclusion, the superinfection is one of the important factors to evolution of virulence by maintenance of genetic diversity, and the spatial structure plays a more important role in a slowly growing plant relative to the speed of spread of the pathogen. In addition, too high a growth rate disadvantages clonal plants, because the rapid growth of the plant assists the spread of the pathogen due to the increase in susceptible individuals, and leads to lower mortality costs of the dominant strain. However, from a biological requirement, we will have to construct and analyze more complicated models to express the dynamics of real plants. In the future, more details will have to be analyzed, such as the bifurcation condition, the values of the equilibrium, and stability of the equilibrium state. In addition, to analyze real pathogens, it is necessary to change the assumption for the mortality cost, such as a more complex relationship between infection rate and virulence, and we will estimate the parameter value, notably difference in morality costs among strains (*Δm*), by comparison between our result and available quantitative data.

## Supporting Information

S1 Appendix Simplification of master equation. (PDF)

S2 Appendix Local stability analysis in mean-field approximation. (PDF)

S3 Appendix Local stability analysis in pair approximation. (PDF)
