# Supplementary material for: Pathogen Propagation Model with Superinfection in Vegetatively Propagated Plants on Lattice Space
Source: PLoS One. 2016 May 5;11(5):e0154883. doi: 10.1371/journal.pone.0154883 (PMC4858194; doi:10.1371/journal.pone.0154883)
Supplement: S1 Appendix — (PDF) [file pone.0154883.s001.pdf]

Simplification of the master equation

The set of Eq. (3) is simplified by the following process: The variables have the following properties from their definitions,

$$P_{\sigma_i\sigma_j} = P_{\sigma_j\sigma_i}, \tag{S1.1}$$

$$\sum_{\sigma_j \in \boldsymbol{S}} P_{\sigma_i\sigma_j} = \rho_{\sigma_i} \ \text{(for any } \sigma_i) , \tag{S1.2}$$

$$\sum_{\sigma_i \in \boldsymbol{S}} \rho_{\sigma_i} = 1, \tag{S1.3}$$

$$\sum_{\sigma_j \in \boldsymbol{S}} q_{\sigma_j/\sigma_i} = 1 \ \text{(for any} \sigma_i) . \tag{S1.4}$$

We obtained the differential equation of each variable from Eqs. (2) and (S1.2),

$$\dot{\rho}_{\sigma_i} = \sum_{\sigma_j \in \boldsymbol{S}} \dot{P}_{\sigma_i\sigma_j}, \tag{S1.5}$$

$$\begin{aligned} \dot{P}_{\sigma_i\sigma_j} &= \dot{\rho}_{\sigma_i} q_{\sigma_j/\sigma_i} + \rho_{\sigma_i} \dot{q}_{\sigma_j/\sigma_i}, \\ \dot{q}_{\sigma_j/\sigma_i} &= \frac{\dot{P}_{\sigma_i\sigma_j} - \dot{\rho}_{\sigma_i} q_{\sigma_j/\sigma i}}{\rho_{\sigma_i}}. \end{aligned} \tag{S1.6}$$

In addition, the following variables were replaced using the remaining variables; i.e. $\left(\rho_{\mathrm{S}}, \rho_{\mathrm{I}}, q_{0/\mathrm{S}}, q_{0/\mathrm{I}}, q_{\mathrm{S}/\mathrm{I}}\right)$ from Eqs. (2), (S1.1), (S1.3), and (S1.4),

$$\begin{aligned} &\rho_0 = 1 - \rho_{\mathrm{S}} - \rho_{\mathrm{I}}, && q_{0/0} = 1 - \frac{\rho_{\mathrm{S}} q_{0/\mathrm{S}}}{1 - \rho_{\mathrm{S}} - \rho_{\mathrm{I}}} - q_{\mathrm{I}/0}, \\ &q_{\mathrm{S}/0} = \frac{\rho_{\mathrm{S}} q_{0/\mathrm{S}}}{1 - \rho_{\mathrm{S}} - \rho_{\mathrm{I}}}, && q_{\mathrm{S}/\mathrm{S}} = 1 - q_{0/\mathrm{S}} - \frac{\rho_{\mathrm{I}} q_{\mathrm{S}/\mathrm{I}}}{\rho_{\mathrm{S}}}, \\ &q_{\mathrm{I}/\mathrm{S}} = \frac{\rho_{\mathrm{I}} q_{\mathrm{S}/\mathrm{I}}}{\rho_{\mathrm{S}}}, && q_{\mathrm{I}/0} = \frac{\rho_{\mathrm{I}} q_{0/\mathrm{I}}}{\left(1 - \rho_{\mathrm{S}} - \rho_{\mathrm{I}}\right)}, \\ &q_{\mathrm{I}/\mathrm{I}} = 1 - \frac{\left(1 - \rho_{\mathrm{S}} - \rho_{\mathrm{I}}\right) q_{\mathrm{I}/0}}{\rho_{\mathrm{I}}} - q_{\mathrm{S}/\mathrm{I}}. \end{aligned} \tag{S1.7}$$

Thus, we obtained the set of equations of five variables from Eqs. (3), (S1.2) and (S1.6), and using PA,

$$
\begin{aligned}
\dot{\rho}_{\mathrm{S}} &= \dot{P}_{0\mathrm{S}} + \dot{P}_{\mathrm{SS}} + \dot{P}_{\mathrm{IS}} \\
&= \rho_{\mathrm{S}} \left(\beta_{\mathrm{S}} q_{0/\mathrm{S}} - m_{\mathrm{I}} q_{\mathrm{I}/\mathrm{S}} \right), \qquad \text{(S1.8)} \\
\dot{\rho}_{\mathrm{I}} &= \dot{P}_{0\mathrm{I}} + \dot{P}_{\mathrm{SI}} + \dot{P}_{\mathrm{II}} \\
&= \rho_{\mathrm{I}} \left(m_{\mathrm{I}} q_{\mathrm{S}/\mathrm{I}} - 1 \right), \qquad \text{(S1.9)} \\
\dot{q_{\mathrm{I}/0}} &= \frac{\dot{P}_{\mathrm{I}0} - \dot{\rho}_0 q_{\mathrm{I}/0}}{\rho_0} \\
&= \frac{\rho_{\mathrm{I}}}{\rho_0} \left(q_{\mathrm{I}/\mathrm{I}} - q_{\mathrm{I}/0} \right) + \frac{\beta_{\mathrm{S}} q_{\mathrm{I}/0} + (z-1)\, m_{\mathrm{I}} q_{\mathrm{I}/\mathrm{S}}}{z} q_{\mathrm{S}/0} - q_{\mathrm{I}/0}, \qquad \text{(S1.10)} \\
\dot{q_{0/\mathrm{S}}} &= \frac{\dot{P}_{0\mathrm{S}} - \dot{\rho}_{\mathrm{S}} q_{0/\mathrm{S}}}{\rho_{\mathrm{S}}} \\
&= q_{\mathrm{I}/\mathrm{S}} \left(\frac{m_{\mathrm{I}} q_{0/\mathrm{S}}}{z} + 1 \right) - \beta_{\mathrm{S}} q_{0/\mathrm{S}} \left(q_{0/\mathrm{S}} + \frac{1 - (z-1) \left(q_{0/0} - q_{\mathrm{S}/0} \right)}{z} \right), \qquad \text{(S1.11)} \\
\dot{q_{\mathrm{S}/\mathrm{I}}} &= \frac{\dot{P}_{\mathrm{SI}} - \dot{\rho}_{\mathrm{I}} q_{\mathrm{S}/\mathrm{I}}}{\rho_{\mathrm{I}}} \\
&= q_{\mathrm{I}/\mathrm{S}} \left[\frac{(z-1)\, m_{\mathrm{I}} \left(q_{\mathrm{S}/\mathrm{S}} - q_{\mathrm{I}/\mathrm{S}} \right) - 1}{z} - m_{\mathrm{I}} q_{\mathrm{S}/\mathrm{I}} \right] + \frac{(z-1)\, \beta_{\mathrm{S}} q_{\mathrm{S}/0}}{z}. \qquad \text{(S1.12)}
\end{aligned}
$$

Therefore, we obtained the set of simplified equations by substituting (S1.7) in Eq. (S1.8)-(S1.12).
