# Supplementary material for: Pathogen Propagation Model with Superinfection in Vegetatively Propagated Plants on Lattice Space
Source: PLoS One. 2016 May 5;11(5):e0154883. doi: 10.1371/journal.pone.0154883 (PMC4858194; doi:10.1371/journal.pone.0154883)
Supplement: S2 Appendix — (PDF) [file pone.0154883.s002.pdf]

Local stability analysis in the mean-field approximation

The Jacobian at Eqs. (4) is:

$$\boldsymbol{J} = \begin{pmatrix} -1-\beta_{\rm S}\rho_{\rm S} & -\beta_{\rm S}\rho_0 - 1 \\ (\beta_{\rm S}+m_{\rm I})\,\rho_{\rm S} & \beta_{\rm S}\rho_0 + m_{\rm I}\rho_{\rm S} - m_{\rm I}\,(1-\rho_0-\rho_{\rm S}) \end{pmatrix}.$$

Extinction region

In the case of the extinction region, the Jacobian is;

$$\hat{\boldsymbol{J}}_{\rm M} \equiv \boldsymbol{J}(\hat{\boldsymbol{E}}_{\rm M}) = \begin{pmatrix} -1 & -\beta_{\rm S}-1 \\ 0 & \beta_{\rm S} \end{pmatrix},$$

thus,

$$Tr(\hat{\boldsymbol{J}}_{\rm M}) = \beta_{\rm S} - 1,\ Det(\hat{\boldsymbol{J}}_{\rm M}) = -\beta_{\rm S}$$

The stability condition ($Tr(\tilde{\boldsymbol{J}}_{siM}) < 0$ and $Det(\tilde{\boldsymbol{J}}_{\rm M}) > 0$) is $0 > \beta_{\rm S}$. Therefore, this equilibrium is always unstable in our assumption.

Disease-free region

In the case of the disease-free region, the Jacobian is;

$$\tilde{\boldsymbol{J}}_{\rm M} \equiv \boldsymbol{J}(\tilde{\boldsymbol{E}}_{\rm M}) = \begin{pmatrix} -1-\beta_{\rm S} & -1 \\ (\beta_{\rm S}+m_{\rm I}) & m_{\rm I} \end{pmatrix},$$

thus,

$$Tr(\hat{\boldsymbol{J}}_{\rm M}) = m_{\rm I} - \beta_{\rm S} - 1,\ Det(\hat{\boldsymbol{J}}_{\rm M}) = \beta_{\rm S}\,(1-m_{\rm I})$$

The stability condition is;

$$0 < \beta_{\rm S},\ m_{\rm I} < 1.$$

Therefore, the stability condition at the equilibrium is $m_{\rm I} < 1$.

Epidemic region

In the case of the epidemic region, the Jacobian is;

$$\bar{\boldsymbol{J}}_{\text{M}} \equiv \boldsymbol{J}(\bar{\boldsymbol{E}}_{\text{M}}) = \begin{pmatrix} -\frac{\beta_{\text{S}}+m_{\text{I}}}{\beta_{\text{S}}} & -\frac{m_{\text{I}}(1+\beta_{\text{S}})}{\beta_{\text{S}}+m_{\text{I}}} \\ \frac{\beta_{\text{S}}+m_{\text{I}}}{\beta_{\text{S}}} & 1 \end{pmatrix},$$

thus,

$$Tr(\hat{\boldsymbol{J}}_{\text{M}}) = -\frac{\beta_{\text{S}}}{m_{\text{I}}},\ Det(\hat{\boldsymbol{J}}_{\text{M}}) = \frac{\beta_{\text{S}}\,(m_{\text{I}}-1)}{m_{\text{I}}}$$

The stability condition is;

$$0 < \beta_{\text{S}},\ 1 < m_{\text{I}}$$

Therefore, the stability condition at the equilibrium is $0 < \beta_{\text{S}}$, $m_{\text{I}} > 1$.
