# Supplementary material for: Pathogen Propagation Model with Superinfection in Vegetatively Propagated Plants on Lattice Space
Source: PLoS One. 2016 May 5;11(5):e0154883. doi: 10.1371/journal.pone.0154883 (PMC4858194; doi:10.1371/journal.pone.0154883)
Supplement: S3 Appendix — (PDF) [file pone.0154883.s003.pdf]

Local stability analysis in pair approximation

To analyze the local stability at each equilibrium state, we used the Routh–Hurwitz stability criterion. Let the characteristic polynomial of the Jacobian of n degrees at equilibrium state be,

$$a_0\lambda^n + a_1\lambda^{n-1} + a_2\lambda^{n-2}\cdots a_{n-1}\lambda + a_n,$$

and the Hurwitz determinant be Δ_n.

Extinction region

In the stability analysis of the extinction equilibrium, we selected the same five variables at S1 and simplified Eqs. (3).

$$\begin{aligned}
\dot{\rho}_{\mathrm{S}} =& \rho_{\mathrm{S}}\left(m_{\mathrm{I}}q_{\mathrm{I/S}} - \beta_{\mathrm{S}}q_{0/\mathrm{S}}\right),\\
\dot{\rho}_{\mathrm{I}} =& m_{\mathrm{I}}\rho_{\mathrm{S}}q_{\mathrm{I/S}} - \rho_{\mathrm{I}},\\
\dot{q}_{\mathrm{I}/0} =& \frac{1}{\left(1-\rho_{\mathrm{S}}-\rho_{\mathrm{I}}\right)z}\left(\rho_{\mathrm{S}}q_{0/\mathrm{S}}\left[\beta_{\mathrm{S}}q_{\mathrm{I}0} + (z-1)\,m_{\mathrm{I}}q_{\mathrm{I/S}}\right]\right.\\
&\left. + \left[\rho_{\mathrm{I}}\left(1+q_{\mathrm{I}/0}\right) - \rho_{\mathrm{S}}q_{\mathrm{I/S}} - \left\{2+(d-2)\,\rho_{\mathrm{S}}\right\}q_{\mathrm{I}/0}\right]z\right),\\
\dot{q}_{0/\mathrm{S}} =& \left(1+\frac{m_{\mathrm{I}}q_{0/\mathrm{S}}}{z}\right)q_{\mathrm{I/S}}\\
&-\beta_{\mathrm{S}}q_{0/\mathrm{S}}\left[q_{0/\mathrm{S}} + \frac{1}{z} - \frac{z-1}{z}\left(\frac{\left(1-\rho_{\mathrm{S}}-\rho_{\mathrm{I}}\right)\left(1-q_{\mathrm{I}/0}\right) - 2\rho_{\mathrm{S}}q_{0/\mathrm{S}}}{1-\rho_{\mathrm{S}}-\rho_{\mathrm{I}}}\right)\right],\\
\dot{q}_{\mathrm{I/S}} =& \frac{1}{z}\left[\beta_{\mathrm{S}}q_{0/\mathrm{S}}\left((z-1)\,q_{\mathrm{I}/0} - q_{\mathrm{I/S}}z\right)\right.\\
&\left. + m_{\mathrm{I}}q_{\mathrm{I/S}}\left((z-2)\left(1-q_{\mathrm{I/S}}\right) - (z-1)\,q_{0/\mathrm{S}}\right)\right] - q_{\mathrm{I/S}}.
\end{aligned} \tag{S3.1}$$

The Jacobian of Eqs. (S3.1) at $\tilde{\boldsymbol{E}}_{\mathrm{P}}$ ($\rho_0^* = 0,\ \rho_{\mathrm{S}}^* = 0,\ q_{\mathrm{I}/0}^* = 0$) is:

$$\begin{aligned}
\tilde{\boldsymbol{J}}_{\mathrm{P}} \equiv& \boldsymbol{J}(\tilde{\boldsymbol{E}}_{\mathrm{P}}),\\
\left|\lambda\boldsymbol{I} - \tilde{\boldsymbol{J}}_{\mathrm{P}}\right| =& (\lambda+2)(\lambda+1)\left(\lambda + m_{\mathrm{I}}q_{\mathrm{I/S}}^* - \beta_{\mathrm{S}}q_{0/\mathrm{S}}^*\right)\\
&\begin{vmatrix}
\lambda + \frac{2\beta_{\mathrm{S}} - m_{\mathrm{I}}q_{\mathrm{I/S}}^*}{z} + -\beta_{\mathrm{S}}(1-2q_{0/\mathrm{S}}^*) & -1 - \frac{m_{\mathrm{I}}q_{0/\mathrm{S}}^*}{z}\\
\frac{((\beta_{\mathrm{S}}+m_{\mathrm{I}})z - m_{\mathrm{I}})q_{\mathrm{I/S}}^*}{z} & \lambda + 1 + \beta_{\mathrm{S}} - \frac{m\left[(z-2)\left(1-2q_{\mathrm{I/S}}^*\right) - (z-1)q_{0/\mathrm{S}}^*\right]}{z}
\end{vmatrix}.
\end{aligned} \tag{S3.2}$$

Two remaining variables have the following equilibrium values.

$$q_{0/\mathrm{S}}^{*} = \frac{m_{\mathrm{I}}\left(z-2\right)-z}{m_{\mathrm{I}}\left(z-1\right)},\ q_{\mathrm{I}/\mathrm{S}}^{*} = \frac{\beta_{\mathrm{S}}\left(m_{\mathrm{I}}\left(z-2\right)-z^{2}\right)\left(m_{\mathrm{I}}\left(z-2\right)-z\right)}{m_{\mathrm{I}}^{2}\left(z-1\right)\left(z-2\right)\left(m_{\mathrm{I}}+z\right)}.$$

Here,

$$\begin{aligned} & m_{\mathrm{I}}q_{\mathrm{I}/\mathrm{S}}^{*}-\beta_{\mathrm{S}}q_{0/\mathrm{S}}^{*} \\ =& -\frac{2\beta_{\mathrm{S}}z\left(m_{\mathrm{I}}\left(z-2\right)-z\right)}{m_{\mathrm{I}}z\left(z-2\right)\left(m_{\mathrm{I}}+z\right)}. \end{aligned} \tag{S3.3}$$

From $q_{0/\mathrm{S}}^{*} > 0$, the Eq. (S3.3) is negative, thus this equilibrium is always unstable.

Disease-free region

In the stability analysis of the disease-free equilibrium, we selected five other variables $\left(\rho_{\mathrm{S}}, \rho_{\mathrm{I}}, q_{0/0}, q_{0/\mathrm{S}}, q_{\mathrm{I}/\mathrm{S}}\right)$ and simplified Eqs. (3).

$$\begin{aligned} \dot{\rho}_{\mathrm{S}} =& \beta_{\mathrm{S}}\rho_{\mathrm{S}}q_{0/\mathrm{S}} - m_{\mathrm{I}}\rho_{\mathrm{I}}q_{\mathrm{S}/\mathrm{I}}, \\ \dot{\rho}_{\mathrm{I}} =& \rho_{\mathrm{I}}\left(m_{\mathrm{I}}q_{\mathrm{S}/\mathrm{I}}-1\right), \\ \dot{q}_{0/0} =& -\frac{1}{z\left(1-\rho_{\mathrm{S}}-\rho_{\mathrm{I}}\right)}\left[\left(z-2\right)\beta_{\mathrm{S}}\rho_{\mathrm{S}}q_{0/0}q_{0/\mathrm{S}}\right. \\ & \left.+z\left(2\rho_{\mathrm{S}}\left(1+q_{0/\mathrm{S}}q_{0/0}\right)-\rho_{\mathrm{I}}q_{0/0}-2\left(1-\rho_{\mathrm{I}}-q_{0/0}\right)\right)\right], \\ \dot{q}_{0/\mathrm{S}} =& \left(1+\frac{m_{\mathrm{I}}q_{0/\mathrm{S}}}{z}\right)q_{\mathrm{I}/\mathrm{S}} - \beta_{\mathrm{S}}q_{0/\mathrm{S}}\left[q_{0/\mathrm{S}}+\frac{1}{z}-\frac{\left(z-1\right)}{z}\left(q_{0/0}-\frac{\rho_{\mathrm{S}}q_{0/\mathrm{S}}}{1-\rho_{\mathrm{S}}-\rho_{\mathrm{I}}}\right)\right], \\ \dot{q}_{\mathrm{I}/\mathrm{S}} =& \frac{\beta_{\mathrm{S}}q_{0/\mathrm{S}}}{z}\left[\left(z-1\right)\left(1-q_{0/0}\right)-zq_{\mathrm{I}/\mathrm{S}}-\frac{\left(z-1\right)\rho_{\mathrm{S}}q_{0/\mathrm{S}}}{1-\rho_{\mathrm{S}}-\rho_{\mathrm{I}}}\right] \\ & +\frac{m_{\mathrm{I}}q_{\mathrm{I}/\mathrm{S}}}{z}\left[\left(z-2\right)\left(1-q_{\mathrm{I}/\mathrm{S}}\right)-\left(z-1\right)q_{0/\mathrm{S}}\right]-q_{\mathrm{I}/\mathrm{S}}. \end{aligned} \tag{S3.4}$$

The Jacobian of Eqs. (S3.4) at $\hat{\boldsymbol{E}}_{\mathrm{P}}$ is:

$$\begin{aligned}
\tilde{\boldsymbol{J}}_{\mathrm{P}} \equiv& \boldsymbol{J}(\tilde{\boldsymbol{E}}_{\mathrm{P}}),\\
\left|\lambda \boldsymbol{I} - \hat{\boldsymbol{J}}_{\mathrm{P}}\right| =& \left(\lambda + 2 + \frac{2\left(z-1\right)\beta_{\mathrm{S}}}{z}\right)\\
&\begin{vmatrix}
\lambda+1 & 0 & 0 & -m_{\mathrm{I}}\\
0 & \lambda & -\beta_{\mathrm{S}} & -1\\
\frac{(z-1)\beta_{\mathrm{S}}}{z} & \frac{(z-1)\beta_{\mathrm{S}}}{z} & \lambda + m_{\mathrm{I}}\left(2-\frac{1}{z}\right) & -1\\
\frac{(z-1)\beta_{\mathrm{S}}}{z} & \frac{(z-1)\beta_{\mathrm{S}}}{z} & \frac{(z-1)\beta_{\mathrm{S}}}{z} & \lambda + 1 - \frac{(z-2)m_{\mathrm{I}}}{z}
\end{vmatrix}.
\end{aligned} \tag{S3.5}$$

Thus, the coefficients of characteristic polynomials and Hurwitz determinants are,

$$\begin{aligned}
a_4 =& \frac{2\beta_{\mathrm{S}}^2\left(z-1\right)\left(z-\left(z-1\right)m_{\mathrm{I}}\right)}{z^2},\\
a_3 =& \frac{\beta_{\mathrm{S}}}{z^2}\left[z\left(3z-2+3\beta_{\mathrm{S}}\left(z-1\right)\right) - m_{\mathrm{I}}\left(z^2\left(a+3\right)-3z\left(a+2\right)+2\left(a+1\right)\right)\right],\\
a_2 =& \frac{z^2\left(1+\beta_{\mathrm{S}}\left(\beta_{\mathrm{S}}+5-2m_{\mathrm{I}}\right)-m_{\mathrm{I}}\right) - z\left(\beta_{\mathrm{S}}\left(\beta_{\mathrm{S}}+3-5m_{\mathrm{I}}\right)-2m_{\mathrm{I}}\right) - 2\beta_{\mathrm{S}}m_{\mathrm{I}}}{z^2},\\
a_1 =& 2\left(\beta_{\mathrm{S}}+1\right) - \frac{\beta_{\mathrm{S}}+\left(z-2\right)m_{\mathrm{I}}}{z},\\
a_0 =& 1,\\
\Delta_2 =& \frac{1}{z^3}\Big[z\left(z-1\right)\left(2z-1\right)\beta_{\mathrm{S}}^3 + \beta_{\mathrm{S}}^2\left\{z\left(3+z\left(9z-10\right)\right)-\left(z-1\right)\left(2z-1\right)^2 m_{\mathrm{I}}\right\}\\
&+\beta_{\mathrm{S}}\left\{z^2\left(9z-5\right)+\left(z-2\right)^2 m_{\mathrm{I}}^2 - 2zm_{\mathrm{I}}\left(5+z\left(4z-11\right)\right)\right\}\\
&+zm_{\mathrm{I}}\left(\left(z-2\right)m_{\mathrm{I}}-2z\right)\left(\left(z-2\right)m_{\mathrm{I}}-z\right)\Big],\\
\Delta_3 =& \frac{\beta_{\mathrm{S}}}{z^5}\left[2m_{\mathrm{I}}\left(\beta_{\mathrm{S}}+1\right)+z\left(2+\beta_{\mathrm{S}}\left(\beta_{\mathrm{S}}+3\right)-m_{\mathrm{I}}\left(5\beta_{\mathrm{S}}+6\right)\right)-z^2\left(2\beta_{\mathrm{S}}+3\right)\left(1+\beta_{\mathrm{S}}-m_{\mathrm{I}}\right)\right]\\
&\Big[\beta_{\mathrm{S}}^2\left(z-1\right)^2\left(\left(z-2\right)m_{\mathrm{I}}-3z\right)-\beta_{\mathrm{S}}\left(z-1\right)\left(\left(z-2\right)^2 m_{\mathrm{I}}^2-2zm_{\mathrm{I}}\left(2z-5\right)+5z^2\right)\\
&-z\left(\left(z-2\right)m_{\mathrm{I}}-2z\right)\left(\left(z-2\right)m_{\mathrm{I}}-z\right)\Big].
\end{aligned}$$

Here, from $a_4 > 0$,

$$m_{\mathrm{I}} < \frac{z}{z-1}, \tag{S3.6}$$

In addition, we confirmed that all the coefficients and determinants are positive under these conditions. Thus, the stability condition of the disease-free equilibrium is Eq. (S3.6).

Epidemic region

In the stability analysis of the epidemic equilibrium, we selected five other variables $\left(\rho_0, \rho_{\mathrm{S}}, q_{0/\mathrm{S}}, q_{0/\mathrm{I}}, q_{\mathrm{S}/\mathrm{I}}\right)$ and simplified Eqs. (3).

$$\dot{\rho}_0 = 1 - \rho_0 - \rho_{\mathrm{S}} \left(1 + \beta_{\mathrm{S}} q_{0/\mathrm{S}}\right), \tag{S3.7}$$

$$\dot{\rho}_{\mathrm{S}} = \beta_{\mathrm{S}} \rho_{\mathrm{S}} q_{0/\mathrm{S}} - m_{\mathrm{I}} \left(1 - \rho_{\mathrm{S}} - \rho_{\mathrm{I}}\right) q_{\mathrm{S}/\mathrm{I}}, \tag{S3.8}$$

$$\begin{aligned}\dot{q}_{0/\mathrm{S}} =& \frac{1}{\rho_0 \rho_{\mathrm{S}}} \left[m_{\mathrm{I}} \rho_0 \left(1 - \rho_0 - \rho_{\mathrm{S}}\right) q_{0/\mathrm{S}} q_{\mathrm{S}/\mathrm{I}} + z \left(1 - \rho_0 - \rho_{\mathrm{S}}\right) \rho_0 q_{\mathrm{S}/\mathrm{I}} \right. \\ & \left. + \beta_{\mathrm{S}} \rho_{\mathrm{S}} q_{0/\mathrm{S}} \left\{ \rho_0 \left(z \left(1 - q_{0/\mathrm{S}}\right) + (z-1) q_{0/\mathrm{I}} - 2 \right) - (z-1) \left(2 \rho_{\mathrm{S}} q_{0/\mathrm{S}} + \left(1 - \rho_{\mathrm{S}}\right) q_{0/\mathrm{I}} \right) \right\} \right],\end{aligned} \tag{S3.9}$$

$$\dot{q}_{0/\mathrm{I}} = 1 + \left(\frac{(z-1) m_{\mathrm{I}} q_{0/\mathrm{S}}}{\rho_{\mathrm{S}}} - 1 \right) q_{\mathrm{S}/\mathrm{I}} - \frac{q_{0/\mathrm{I}} \left(z \rho_0 \left(1 + m_{\mathrm{I}} q_{\mathrm{S}/\mathrm{I}}\right) + (z-1) \beta_{\mathrm{S}} \rho_{\mathrm{S}} q_{0/\mathrm{S}} \right)}{z \rho_0}, \tag{S3.10}$$

$$\begin{aligned}\dot{q}_{\mathrm{S}/\mathrm{I}} =& \frac{1}{\rho_0 \rho_{\mathrm{S}}} \left[(z-1) \beta_{\mathrm{S}} \rho_{\mathrm{S}}^2 q_{0/\mathrm{S}} q_{0/\mathrm{I}} \right. \\ & \left. + \rho_0 q_{\mathrm{S}/\mathrm{I}} \left\{ m_{\mathrm{I}} \left(\rho_{\mathrm{S}} \left[(z-2) \left(1 + q_{\mathrm{S}/\mathrm{I}}\right) - (z-1) q_{0/\mathrm{S}} \right] - 2 (z-1) \left(1 - \rho_0\right) q_{\mathrm{S}/\mathrm{I}} \right) \right\} \right].\end{aligned} \tag{S3.11}$$

From above equations (Eqs. (S3.7)-(S3.11)), we derived following equilibrium value, and all other variables were derived by Eq. (**??**).

$$\begin{aligned}
\bar{\rho}_{\mathrm{S}}^* =& \frac{z \left(m_{\mathrm{I}} - 1\right) + (z-1) m_{\mathrm{I}} - 2 z m_{\mathrm{I}} \bar{q}_{0/\mathrm{I}}^*}{\left[z \left(m_{\mathrm{I}} - 1\right) + (z-1) m_{\mathrm{I}} \bar{q}_{0/\mathrm{S}}^* \right] \left(1 + \beta_{\mathrm{S}}\right) - \left[(z-1) \beta_{\mathrm{S}} \bar{q}_{0/\mathrm{S}}^* + 2z \right] m_{\mathrm{I}} \bar{q}_{0/\mathrm{I}}^*}, \\
\bar{\rho}_{\mathrm{I}}^* =& \beta_{\mathrm{S}} \bar{\rho}_{\mathrm{S}}^* \bar{q}_{0/\mathrm{S}}^*, \\
\bar{q}_{0/\mathrm{S}}^* =& \frac{z m_{\mathrm{I}} \left(1 - \bar{q}_{0/\mathrm{I}}^*\right) - \left(z + m_{\mathrm{I}}\right)}{(z-1) \beta_{\mathrm{S}}}, \\
\bar{q}_{\mathrm{S}/\mathrm{I}}^* =& \frac{1}{m_{\mathrm{I}}}, \\
\bar{q}_{0/\mathrm{I}}^* =& \frac{1}{2 z \beta_{\mathrm{S}} m_{\mathrm{I}} \left(\beta_{\mathrm{S}} + m_{\mathrm{I}}\right)} \left(\beta_{\mathrm{S}}^2 \left(m_{\mathrm{I}} - z\right) + \beta_{\mathrm{S}} m_{\mathrm{I}}^2 (z-1) - z m_{\mathrm{I}} \left(3 \beta_{\mathrm{S}} + m_{\mathrm{I}}\right) \right. \\
& + \sqrt{4 z \beta_{\mathrm{S}} \left(\beta_{\mathrm{S}} + m_{\mathrm{I}}\right) \left[(z-1) m_{\mathrm{I}}^3 + z m_{\mathrm{I}} \left(\beta_{\mathrm{S}} m_{\mathrm{I}} - \beta_{\mathrm{S}} - m_{\mathrm{I}}\right) \right]} \\
& \overline{ + \left(z m_{\mathrm{I}} \left(3 \beta_{\mathrm{S}} + m_{\mathrm{I}}\right) - (z-1) \beta_{\mathrm{S}} m_{\mathrm{I}}^2 - \left(m_{\mathrm{I}} - z\right) \beta_{\mathrm{S}}^2 \right)^2 } \left. \right).
\end{aligned}$$

Here, we abbreviated the Jacobian at equilibrium $\bar{\boldsymbol{E}}_{\mathrm{P}}$, the coefficients of characteristics polynomials and Hurwitz determinants, because these are too long to write in this paper. In addition, it is too difficult to derive the stability condition analytically, thus

we confirmed the value of coefficients and determinants numerically. As a result, all coefficients, Δ_2 and Δ_3 are always positive, and the sign of Δ_4 varies depending on parameter values, in other words, the Hopf bifurcation occurs to exceed parameter values at threshold.
